# Association of the TGFβ gene family with microenvironmental features of gastric cancer and prediction of response to immunotherapy

**DOI:** 10.3389/fonc.2022.920599

**Published:** 2022-09-02

**Authors:** Bangling Han, Tianyi Fang, Yao Zhang, Yongle Zhang, Jialiang Gao, Yingwei Xue

**Affiliations:** Department of Gastrointestinal Surgery, Harbin Medical University Cancer Hospital, Harbin, China

**Keywords:** TGFβ, tumor microenvironment, epithelial-mesenchymal transition, hypoxia, immunotherapy, gastric cancer

## Abstract

In the complex tumor microenvironment, TGFβ is a pleiotropic cytokine involved in regulating cellular processes such as cancer cell proliferation, apoptosis and metastasis. TGFβ defines three subtypes (TGFβ1, TGFβ2, and TGFβ3), of which TGFβ is highly expressed in many cancers, especially those showing high dissemination potential. In addition, increased expression of TGFβ in multiple cancers is usually positively correlated with epithelial mesenchymal transition (EMT) and coordinated with the expression of genes driving EMT-related genes. TGFβ signaling in the tumor microenvironment inhibits the antitumor function of multiple immune cell populations, including T cells and natural killer cells, and the resulting immunosuppression severely limits the efficacy of immune checkpoint inhibitors and other immunotherapeutic approaches. As a major pathway to enhance the efficacy of cancer immunotherapy effects, the role of TGFβ signaling inhibitors have been evaluated in many clinical trials. However, the potential functions and mechanisms of TGFβ1, TGFβ2 and TGFβ3 in gastric cancer progression and tumor immunology are unclear. In this study, we comprehensively analyzed TGFβ1, TGFβ2 and TGFβ3 and gastric cancer microenvironmental features, including immune cell infiltration, EMT, hypoxia, mutation, immunotherapy and drug treatment, based on HMUCH sequencing data (GSE184336) and public databases. We also validated the protein expression levels of TGFβ in gastric cancer tissues as well as the role of TGFβ factor in cytology experiments. This report reveals the important role of the TGFβ gene family in gastric cancer and provides possible relationships and potential mechanisms of TGFβ in gastric cancer.

## Introduction

In the past decades, great progress has been made in surgical treatment techniques and adjuvant therapy for gastric cancer, but its prognosis is still not ideal, and cancer recurrence often occurs (1, 2). With the development of tumor biology, more and more studies have shown that the occurrence, development and metastasis of tumors are closely related to the tumor microenvironment (TME). TME is both the cause and the result of tumor development, therefore, understanding the characteristics of TME and their changing features at different stages of tumor development is of great significance for tumor diagnosis and treatment.

TGFβ is a powerful cytokine in the tumor microenvironment that regulates most cellular behaviors in the TME. In general, TGFβ enhances immune tolerance and suppresses inflammation, mechanisms that are often exploited during tumor evolution to evade surveillance and combat by the immune system (3). In addition, TGFβ molecules can also play an important role in promoting EMT, phenotypic transformation of CAFs, angiogenesis and maintaining tumor stemness in tumors (4–7). The close link between TGFβ signaling pathway and tumor development makes TGFβ signaling pathway a possible new target for tumor therapy. As a new therapeutic strategy, a growing number of drugs aim to block the activation of TGFβ signaling, including TGFβ isoform-specific blocking antibodies, given the favorable toxicity profile of these TGFβ inhibitors, as well as their ability to modulate immune checkpoint activity, TGFβ Inhibitors can synergistically enhance the efficacy of various immunotherapies (8, 9).

Currently, research on the TGFβ molecule family (TGFβ1, TGFβ2 and TGFβ3) has focused on TGFβ1 and TGFβ2 molecules, while studies related to TGFβ3 molecules are still relatively rare (10–13). To comprehensively analyze the potential functions and roles of the TGFβ molecular family in gastric cancer, we performed a comprehensive analysis of TGFβ in HMUCH sequencing data (GSE184336) and multiple gastric cancer public datasets, and validated it in gastric cancer tissues and gastric cancer cells.

## Materials and methods

### Sample collection and data collection

We collected frozen tissues from 231 patients who underwent radical gastric cancer surgery from 2016 to 2019 at the Harbin Medical University Cancer Hospital. Inclusion criteria were preoperative CT examination or gastroscopy and pathological examination to confirm gastric cancer, excluding patients with preoperative chemotherapy, radiotherapy and other adjuvant treatments. All gastric cancer tissues were examined independently by two certified pathologists to confirm the histological type. High-throughput sequencing data of the transcriptome of gastric cancer samples were uploaded to the GEO dataset (GSE184336). This study complied with the requirements of the Research Ethics Committee of the Affiliated Cancer Hospital of Harbin Medical University (2019-164-R).

In addition, gene expression data for pan-cancer were downloaded from the public database The Cancer Genome Atlas (TCGA), which for gastric adenocarcinoma (STAD) also includes copy number variants, mutation data (MAF) and corresponding clinicopathological data. In the TNM staging system, T refers to the condition of the primary tumor site and the extent of adjacent tissue involvement, N refers to regional lymph node involvement, and M refers to distant metastasis. Abbreviations for Pan-cancer showed in **Supplementary Table 1**. The count data used for differential analysis of genes between different groups, and TPM data used to compare the expression levels of different genes. Download the GSE15459, GSE26253, GSE29272, GSE34942, GSE62254, GSE63089 and GSE84437 gastric cancer datasets from the Gene Expression Omnibus (GEO) database for further analysis (14–20).

### Gene set variation analysis

In the GSE184336 dataset we performed GSVA calculations using the R package “GSVA” (21). GSVA is a non-parametric and unsupervised algorithm commonly used to estimate changes in pathway and biological process activity in samples of expression datasets. The “c2.cp.kegg.v7.1.symbols” and “h.all.v7.1.entrez.gmt” gene sets were downloaded from the MSigDB database (http://www.gsea-msigdb.org/)and used to run GSVA. The optimal cutoff values of TGFβ1, TGFβ2 and TGFβ3 were determined according to the ROC curve and grouped. The limma package was used to analyze enrichment score (ES) matrices between different TGFβ subgroups to explore biological differences between patients in different TGFβ subgroups.

### Gastric cancer microenvironment assessment

To assess the gastric cancer microenvironment, the stromalscore, immunescore and ESTIMATEScore were calculated using the ESTIMATE package, where tumor purity = cos(0.6049872018 + 0.0001467884 * ESTIMATEScore) (22). The ssGSEA algorithm assessed the EMT scores and the composition of different types of immune cells in patients with gastric cancer (18, 23).

### Mutation analysis

Gistic 2.0 software was used to identify somatic copy number changes (24). The R package maftools was used to analyze MAF mutation information (25). The SCNA module in the TIMER database (http://timer.cistrome.org/) compared the relationship between different somatic copy number changes of TGFβ and immune cell infiltration (26).

### Prediction of immunotherapy and chemotherapy

In this study, we used the submap module in the GenePattern cloud server (https://cloud.genepattern.org/gp) to analyze the response of patients with different TGFβ subgroups to immunotherapy (PD-1 and CTLA-4) (27). In addition, we also used the ImmuCellAI database (http://bioinfo.life.hust.edu.cn/ImmuCellAI) to predict the response of immune checkpoint blockade (ICB) therapy (anti-PD1 or anti-CTLA4 therapy) (28). Patient sensitivity to drugs was assessed according to the Genomics of Drug Sensitibity in Cancer (GDSC) database, and the half maximal inhibitory concentration (IC50) was quantified and analyzed by the R package pRRophetic (29).

### Western blot analysis and immunohistochemistry

Six pairs of frozen samples of gastric cancer and adjacent normal tissues (more than 5 cm from the tumor margin) were selected, and total protein was extracted with lysis buffer containing protease inhibitors, and the concentration was determined. PVDF membranes (Merck Millipore) were blocked with 5% skim milk powder and incubated overnight at 4°C with primary antibodies (TGFβ1, AF1027; TGFβ2, 19999-1-AP and TGFβ3, 18942-1-AP). Gastric cancer tissue was paraffin-embedded and cut into 5 mm thick sections, and immunohistochemistry (IHC) staining was performed as previously described (30).

### Conditional culture of gastric cancer cells

Gastric cell line (HGC-27) was purchased from Shanghai Institutes for Biological Sciences, Chinese Academy of Sciences, and cultured at 37°C in RPMI-1640 medium containing 10% 100 U/ml penicillin and 100 U/ml streptomycin. The active recombinant proteins TGFβ1 (Proteintech, HZ-1011), TGFβ2 (Proteintech, HZ-1092) and TGFβ3 (Proteintech, HZ-1090) were solubilized and added to normal medium to adjust the concentration of TGFβ factor to 10ng/mL. The gastric cancer cells cultured with TGFβ active protein were added as the TGFβ-treated group, and human serum albumin (HSA) of equal quality was added as the control group and incubated in the cell incubator for 48 hours.

### Transient transfection

HGC27 cells were inoculated in 6-well plates and when the cell growth density reached 40-50%, transfection was performed using jetPRIME transfection reagent (Polyplus Transfection, France) according to the manufacturer’s protocol. siRNA sequences were shown in **Supplementary Table 2**.

### qRT-PCR and transwell assay

Total RNA was extracted from GC cell lines using TRIzol (Transgen Biotechnology, China). RNA was reverse-transcribed into cDNA using reverse transcription system (Promega, USA). The SYBR-Green (Vazyme) mixed system was then assayed in a LightCycler^®^ 480 Real-Time PCR System (Roche) to analyze the cDNA expression levels. The 2-ΔΔCt method was used to calculate the relative expression levels (31). Transwell was applied based on the previous method (32). The PCR primers were shown in **Supplementary Table S3**.

### Statistical analysis

The chi-square test was used to analyze the association between different TGFβ subgroups and clinicopathological parameters. Kaplan-Meier (KM) survival curves were used to compare the survival analysis of different subgroups, and univariate cox regression analysis was also performed on TGFβ. Pearson correlation analysis was performed to test the relationship between the different variables. The results of the experimental data were expressed as mean ± SD (standard deviation). Statistical analyses were performed in R software (https://www.r-project.org/, version 4.01) and Graphpad Pris 7.0. The P-value < 0.05 was considered statistically significant.

## Results

### Gene characteristics and expression levels of TGFβ

**Figure 1:**
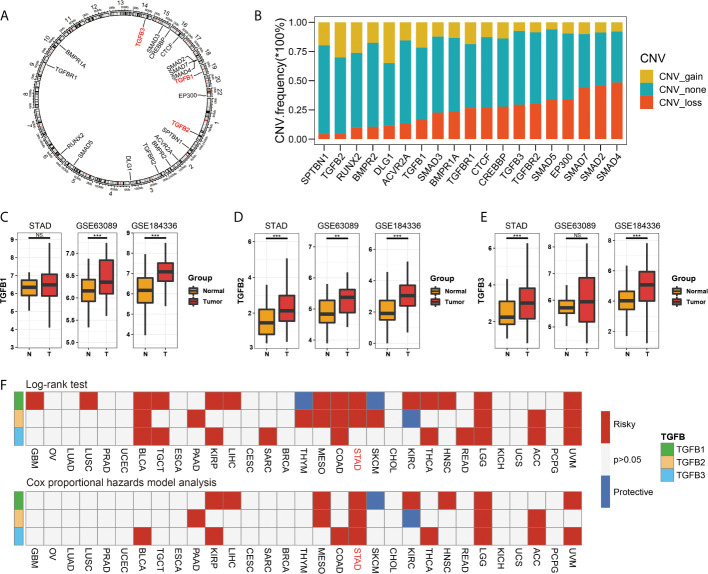
TGFβ gene characteristics. The chromosomal location of the TGFβ gene family and related genes **(A)**, and compared the copy number changes (gain, none and loss) of different genes **(B)**. Comparison of expression levels of TGFβ1, TGFβ2 and TGFβ3 in cancer and paracancerous tissues in STAD, GSE63089 and GSE184336 data sets **(C–E)**. Cox univariate analysis and Kaplan Meier survival analysis of TGFβ1, TGFβ2 and TGFβ3 in pan–cancer **(F)**. *P < 0.05; **P < 0.01; ***P < 0.001; ns, no significance.

We first showed the chromosomal location of the TGFβ gene family and related genes (GeneMANIA database, https://genemania.org/), and compared the copy number changes (gain, none and loss) of different genes (**Figures 1A, B**). In gastric cancer dataset STAD, GSE63089 and dataset GSE184336, the expression levels of TGFβ1, TGFβ2 and TGFβ3 were higher in cancer tissues than in normal tissues adjacent to the cancer (**Figures 1C–E**).

### Survival analysis of TGFβ in pan-cancer and gastric cancer

The results of the Cox univariate analysis in the TCGA database showed that TGFβ1, TGFβ2 and TGFβ3 were prognostic risk factors in most cancers (BLCA, PAAD, KIRP, LIHC, MESO, COAD, THCA, HNSC, LGG, ACC and UVM), notably TGFβ1, TGFβ2 and TGFβ3 were all poor prognostic factors in STAD. The KM survival analysis results showed that patients with high expression levels of TGFβ1, TGFβ2 and TGFβ3 (GBM, LUSC, TGCT, SARC, BLCA, PAAD, KIRP, LIHC, MESO, COAD, THCA, HNSC, READ, LGG, ACC and UVM) had a shorter survival (**Figure 1F**).

**Figure 2:**
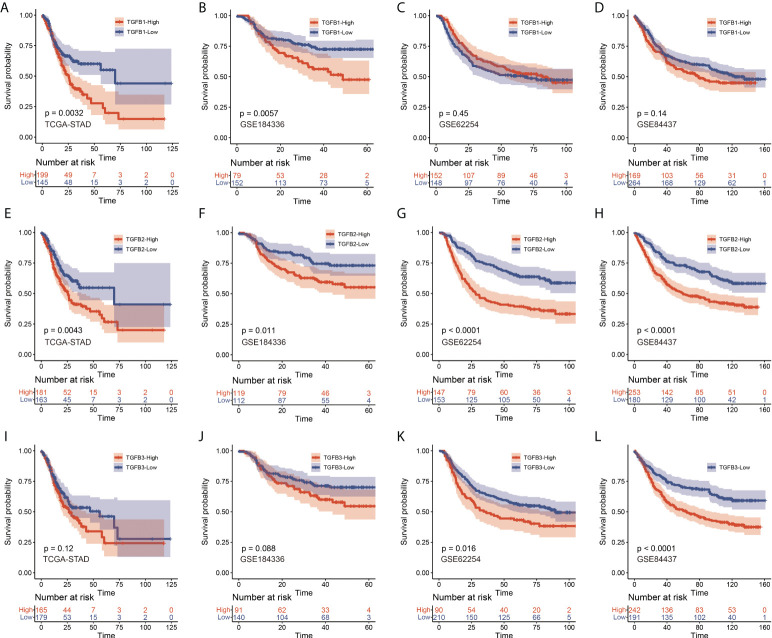
KM survival analysis of TGFβ1 **(A–D),** TGFβ2 **(E–H)** and TGFβ3 **(I–L)** in STAD, GSE184336, GSE84437 and GSE62254 data sets, respectively.

We also performed KM survival analysis on the expression levels of TGFβ in multiple gastric cancer datasets, and the results showed that patients with high expression of TGFβ1 in STAD and GSE184336 had shorter survival time (**Figures 2A–D**), patients with high expression of TGFβ2 in STAD, GSE184336, GSE84437 and GSE62254 had shorter survival times (**Figures 2E–H**), and patients with high expression of TGFβ3 in GSE84437 and GSE62254 had shorter survival time (**Figures 2I–L**). The above results indicated that the high expression of TGFβ in gastric cancer patients was an unfavorable factor for survival.

### Detection of protein level of TGFβ in gastric cancer tissue

**Figure 3:**
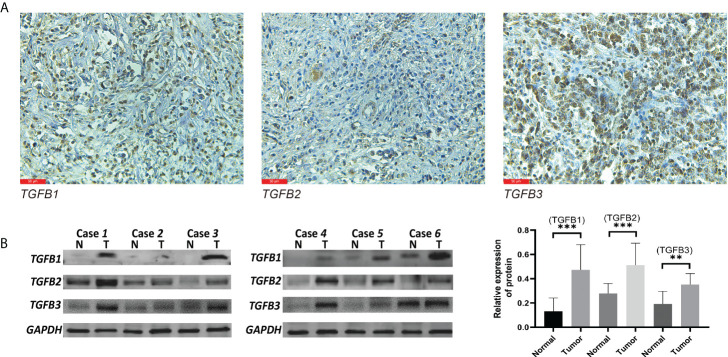
TGFβ protein level detection. Western blot **(A)** and IHC **(B)** validation of TGFβ1, TGFβ2 and TGFβ3 proteins in gastric cancer tissues. **P < 0.01; ***P < 0.001.

We further examined the distribution and expression of TGFβ in gastric cancer tissues by IHC and Western blot assays. Firstly, immunohistochemical experiments were performed on paraffin sections of gastric cancer to observe the main distribution of TGFβ1, TGFβ2 and TGFβ3 proteins in gastric cancer tissues. TGFβ1, TGFβ2, and TGFβ3 protein expressions were distributed in similar regions, with higher levels in the cell cytoplasm, and a small amount in the tumor stroma (**Figure 3A**). Tissue proteins from gastric cancer and normal tissues adjacent to the cancer were extracted, and the results of Western blot experiments showed that the protein expression levels of TGFβ1, TGFβ2 and TGFβ3 were higher in gastric cancer tissues than in normal tissues, which was consistent with the results of transcriptome sequencing levels (GSE184336) (**Figure 3B**).

### Relationship between different TGFβ expression and clinicopathological factors in gastric cancer

TGFβ expression was closely related to clinicopathological factors. TGFβ1 subgroups were associated with TNM, T and N staging in GSE184336 and with T and Histologic Grade in STAD (**Supplementary Table 4**). However, the clinicopathological characteristics of patients between different TGFβ2 expression subgroups were not statistically significant (**Supplementary Table 5**). TGFβ3 subgroups correlated with TNM, T and N stages in GSE184336 and with TNM, T and Histologic Grade in STAD (**Supplementary Table 6**). The combined statistical results showed that high TGFβ expression was associated with poor pathological staging, such as more advanced tumor stage and poorer tumor grading.

### TGFβ-related gene network and TGFβ signaling pathway

The GeneMANIA database was used for protein-protein interaction (PPI) network analysis of TGFβ and related genes, and the KEGG database (Kyoto Encyclopedia of Genes and Genomes, https://www.kegg.jp/kegg/) demonstrated the regulatory network of the TGFβ signaling pathway (TGF-beta signaling pathway - Homo sapiens (human)), and the results showed that the TGFβ signaling pathway may also be involved in apoptosis and cell cycle regulation (**Supplementary Figure 1**).

### Functional enrichment analysis in different TGFβ groupings

**Figure 4:**
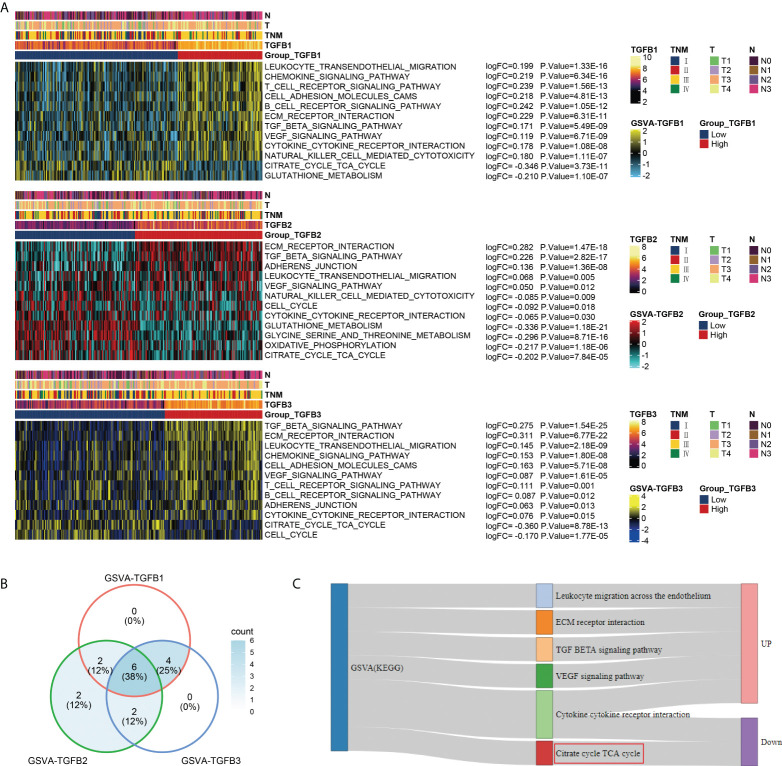
Functional enrichment analysis of different TGFβ groupings. **(A)** Heatmaps were used to visualize biological processes across different TGFβ1, TGFβ2 and TGFβ3 groupings. **(B)** Venn diagram showing the intersection of differential functional pathways between different TGFβ groupings in the dataset GSE184336. **(C)** Sankey diagram showing the expression of 6 intersecting pathways.

In the GSE184336 dataset, we showed the functional expression (KEGG) matrix among different TGFβ1, TGFβ2 and TGFβ3 groupings, respectively (**Figure 4A**). The results showed a strong consistency in the functions involved in TGFβ1, TGFβ2 and TGFβ3 expression in different grouping situations (**Figure 4B**). Among the functional regulation of different TGFβ factors, high expression of TGFβ1, TGFβ2 and TGFβ3 are all involved in promoting Leukocyte migration across the endothelium, ECM receptor interaction, TGF BETA signaling pathway, and VEGF signaling pathway. However, in contrast, high expression of TGFβ1, TGFβ2 and TGFβ3 all inhibited Citrate cycle (TCA cycle) (**Figure 4C**).

### TGFβ and hypoxia

**Figure 5:**
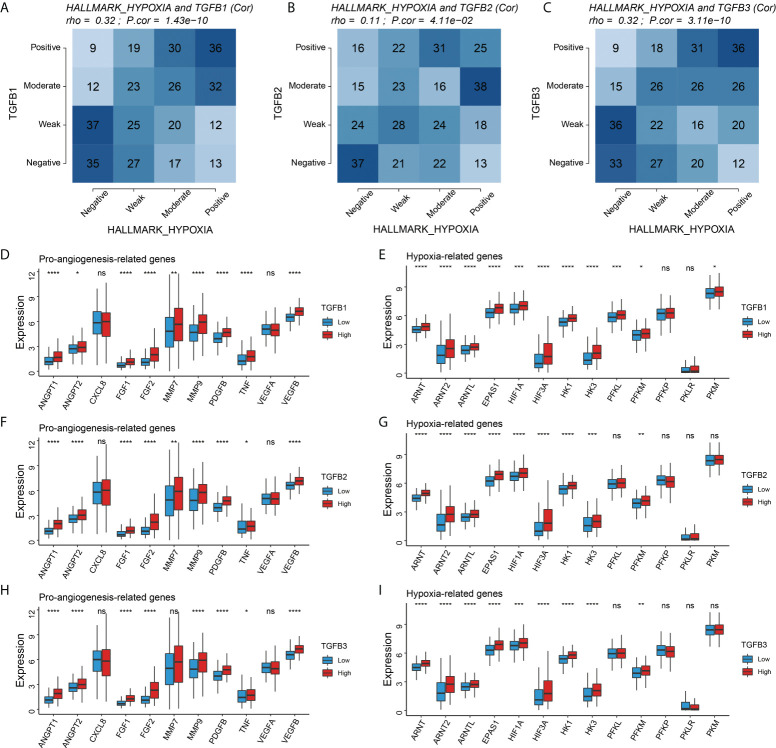
Correlation of TGFβ with hypoxia. **(A–C)** Correlation analysis of TGFβ1, TGFβ2 and TGFβ3 with Hallmark hypoxia, respectively. Comparison of pro–angiogenesis–related genes and hypoxia–related genes among different TGFβ1 **(D, E),** TGFβ2 **(F,G)**, and TGFβ3 **(H, I)** subgroups. *P< 0.05; **P < 0.01; ***P < 0.001; ****P < 0.0001. ns, no significance.

Given the important role of the TCA cycle in energy supply, its physiological processes are susceptible to the influence of the oxygen environment. Therefore, we further analyzed the relationship of TGFβ1, TGFβ2 and TGFβ3 with hypoxia in the STAD dataset. The Hallmark hypoxia score was calculated with reference to the “h.all.v7.1.entrez.gmt” gene set to evaluate the hypoxia level of patients. Correlation analysis showed that the expressions of TGFβ1, TGFβ2 and TGFβ3 were significantly positively correlated with Hallmark hypoxia (**Figures 5A–C**). In addition, hypoxia-related genes (ARNT, ARNT2, ARNTL, EPAS1, HIF1A, HIF3A, HK1, HK3 and PFKM) and pro-angiogenesis-related genes (ANGPT1, ANGPT2, FGF1, FGF2, MMP9, PDGFB, TNF and VEGFB) were highly expressed in the high TGFβ1, TGFβ2 and TGFβ3 expression subgroups (**Figures 5D–I**). Therefore, hypoxia may be one of the important factors that TGFβ participates in promoting gastric cancer progression.

### TGFβ and stromalscore, immunescore and tumor purity

**Figure 6:**
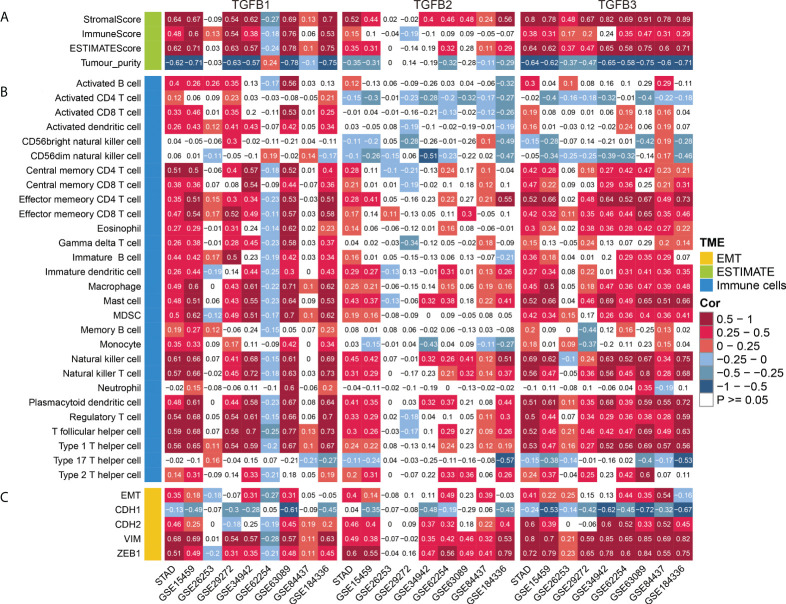
TGFβ and TME. **(A)** Correlation analysis of TGFβ and stromalscore, immunescore, ESTIMATEScore and tumor purity. **(B)** Correlation analysis of TGFβ and immune cells. **(C)** Correlation analysis of TGFβ and EMT, CDH1, CDH2, VIM and ZEB1 in multiple gastric cancer datasets.

Analysis of multiple gastric cancer data showed higher stromalscore and immunescore levels and lower tumor purity with high TGFβ expression (**Figure 6A**). Therefore, in the gastric cancer microenvironment, TGFβ may be involved in promoting increased mesenchymal components and immune cell infiltration.

### Correlation of TGFβ with immune cells

TGFβ is a major regulator of multiple immune cell functions. To investigate the relationship between TGFβ and immune cell infiltration in gastric cancer, we analyzed the correlation between TGFβ and multiple immune cells. As shown in the figure, TGFβ1, TGFβ2 and TGFβ3 were significantly positively correlated with immune cell infiltration in multiple gastric cancer datasets. It is worth noting that due to the strong heterogeneity among gastric cancers, TGFβ1 was negatively correlated with the infiltration level of most immune cells in GSE62254. In addition, a small number of immune cells such as Activated CD4 T cells, Activated CD8 T cells and CD56dim natural killer cells were negatively correlated with TGFβ2 expression (**Figure 6B**). As a whole, TGFβ effectively promoted immune cell infiltration in the gastric cancer microenvironment.

We further analyzed the correlation of TGFβ with multiple immune cell marker genes using the TIMER database (https://cistrome.shinyapps.io/timer/), with correlation options including none and tumor purity (**Supplementary Table 7**). The results showed that TGFβ1, TGFβ2 and TGFβ3 were significantly positively correlated with most immune cell marker genes, including CD8+ T cell, B cell, Monocyte, TAM, M1 Macrophage, M2 Macrophage, Treg, T cell, Neutrophils, Dendritic cell, Th1, Th2, Tfh, Th17 and T cell exhaustion.

### TGFβ and EMT

**Figure 7:**
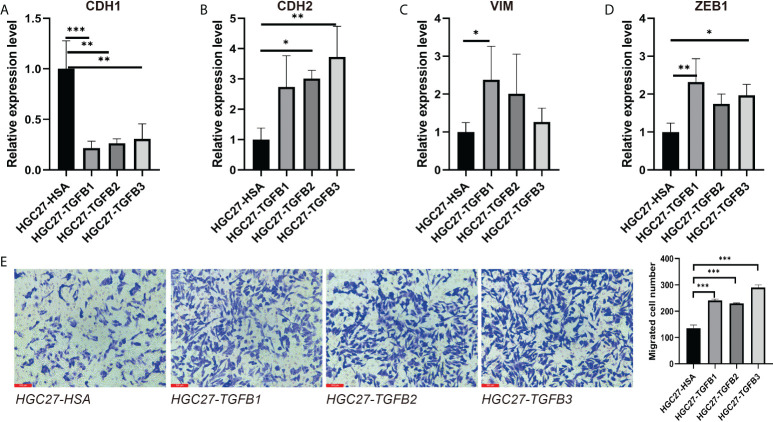
Induction of gastric cancer cells under TGFβ environment. **(A–D)** CDH1, CDH2, VIM and ZEB1 expression in gastric cancer cells in the context of TGFβ. **(E)** The migration ability of gastric cancer cells in TGFβ experimental group and control group was detected by transwell. *P < 0.05; **P < 0.01; ***P < 0.001.

In multiple gastric cancer datasets, TGFβ were positively correlated with EMT score and mesenchymal markers (CDH2, VIM and ZEB1), while significantly negatively correlated with epithelial marker CDH1 (**Figure 6C**). To verify the promoting effect of TGFβ on EMT, we added TGFβ1, TGFβ2 and TGFβ3 active proteins to the culture medium, respectively, and cultured gastric cancer cells in TGFβ environment to detect the changes of cancer cell phenotype. After 48 hours of culture, RNA was extracted from the samples. qRT-PCR results showed that CDH1 expression was decreased and CDH2, VIM and ZEB1 expression were significantly increased in the TGFβ experimental group compared with the control group (**Figures 7A–D**), which indicated that TGFβ is an important regulator of EMT and the promotion of EMT progression in gastric cancer cells.

**Figure 8:**
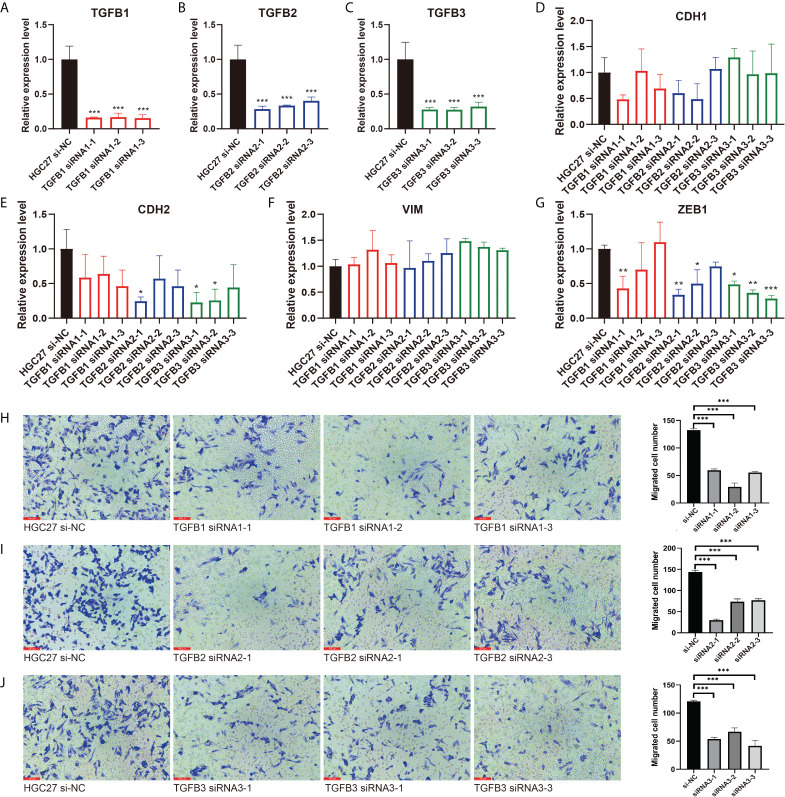
Inhibition of EMT and migration progression after silencing of TGFβ. **(A–C)** The TGFβ1, TGFβ2 and TGFβ3 silencing group was compared with the HGC27 normal group, respectively. **(D–G)** qRT–PCR experiments showed the changes of EMT–related markers CDH1, CDH2, VIM and ZEB1 after TGFβ silencing. **(H–J)** Detection of migration ability of gastric cancer cells in TGFβ–silenced group and control group. *P < 0.05; **P < 0.01; ***P < 0.001.

In view of the important role of TGFβ in EMT, we used small interfering RNA (siRNA) to reduce TGFβ1, TGFβ2 and TGFβ3 gene expression in gastric cancer cells. 48 hours after transfection, we extracted RNA from all samples and qRT-PCR results showed that TGFβ1, TGFβ2 and TGFβ3 expression levels were significantly reduced (**Figures 8A–C**). We continued the qRT-PCR analysis of the samples, and among the EMT-related markers, the expression levels of mesenchymal markers CDH2 and ZEB1 genes were significantly reduced in gastric cancer cells after TGFβ gene silencing, while CDH1 expression was not significantly altered (**Figures 8D–G**). Thus, after silencing the TGFβ gene in gastric cancer cells, the progression of EMT was significantly inhibited, and all of these results together suggest that TGFβ1, TGFβ2 and TGFβ3 are key factors in regulating the progression of EMT.

### TGFβ promoted gastric cancer cell migration

We further explored the alteration of TGFβ on the growth and function of gastric cancer cells. Transwell assay examined the change of gastric cancer cell migration ability in TGFβ experimental group and control group (HSA), and the migration ability of gastric cancer cells was enhanced under TGFβ1, TGFβ2 and TGFβ3 active protein stimulation conditions (**Figure 7E**). In contrast, the migration ability of gastric cancer cells was reduced and statistically significant after silencing of TGFβ1, TGFβ2 and TGFβ3 genes (**Figures 8H–J**). Therefore, TGFβ has a strong promoting effect on cancer cell value addition and migration. Considering the significant suppression of EMT trend after TGFβ gene silencing, EMT may play an indispensable role in the regulation of gastric cancer cell migration by TGFβ.

### TGFβ with TMB and MATH

**Figure 9:**
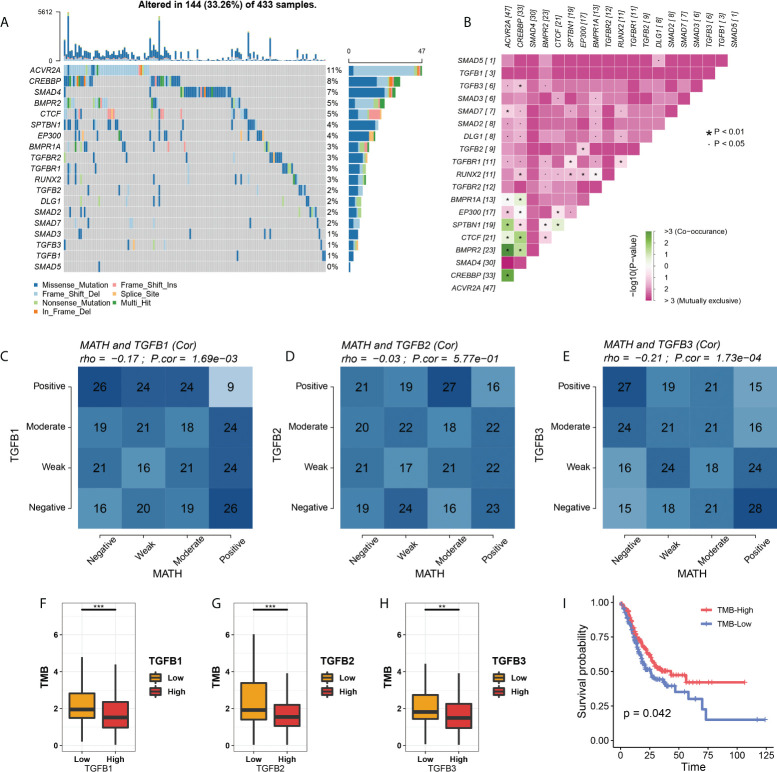
TGFβ with MATH and TMB. **(A)** Waterfall plot of TGFβ–related gene mutations. **(B)** Exclusive and Co–occurrence analysis of pairwise mutant genes. **(C–E)** Correlation analysis of TGFβ and MATH. **(F–H)** Comparison of TMB among different TGFβ groups. **(I)** Kaplan–Meier survival analysis of TMB in STAD. *P < 0.05; **P < 0.01; ***P < 0.001.

We first showed the mutation information of TGFβ and related genes, among which ACVR2A, CREBBP, SMAD4, BMPR2 and CTCF had higher mutation frequencies. Mutation exclusive and co-occurrence among mutant genes were mainly concentrated in genes with high mutation frequency (ACVR2A, CREBBP, SMAD4, BMPR2, CTCF, SPTBN1, EP300 and BMPR1A) (**Figures 9A, B**). In addition, TGFβ1 and TGFβ3 expression were negatively correlated with Mutant-allele tumor heterogeneity (MATH) (**Figures 9C–E).** We also compared the tumor mutation burden (TMB) between different TGFβ groups and showed that TMB was higher in the TGFβ1, TGFβ2 and TGFβ3 low expression groups (*P* < 0.05), while higher TMB was beneficial for prolonging patient survival (**Figures 9F–I**). Therefore, the high expression of TGFβ in the gastric cancer microenvironment may be an important factor in promoting tumor differentiation and poor prognosis.

### Analysis of copy number variation between different TGFβ groups and immune cell infiltration

We showed the differences in copy number variants (CNV) in different TGFβ groupings (**Supplementary Figures 2A, C, E**) and also analyzed the effect of somatic copy number alterations (SCNAs) of TGFβ on immune cell infiltration to elucidate the potential mechanism of TGFβ associated with immune cell infiltration. Arm-level deletion in TGFβ1-associated SCNAs was significantly associated with the level of B-cell, CD4+ T-cell, CD8+ T-cell, neutrophil, macrophage, and dendritic cell infiltration. In contrast, arm-level gain in TGFβ2- and TGFβ3-related SCNAs had a greater effect on B cells, CD4+ T cells, CD8+ T cells, neutrophils, macrophages and dendritic cell infiltration (**Supplementary Figures 2B, D, F**).

### TGFβ and immunotherapy

**Figure 10:**
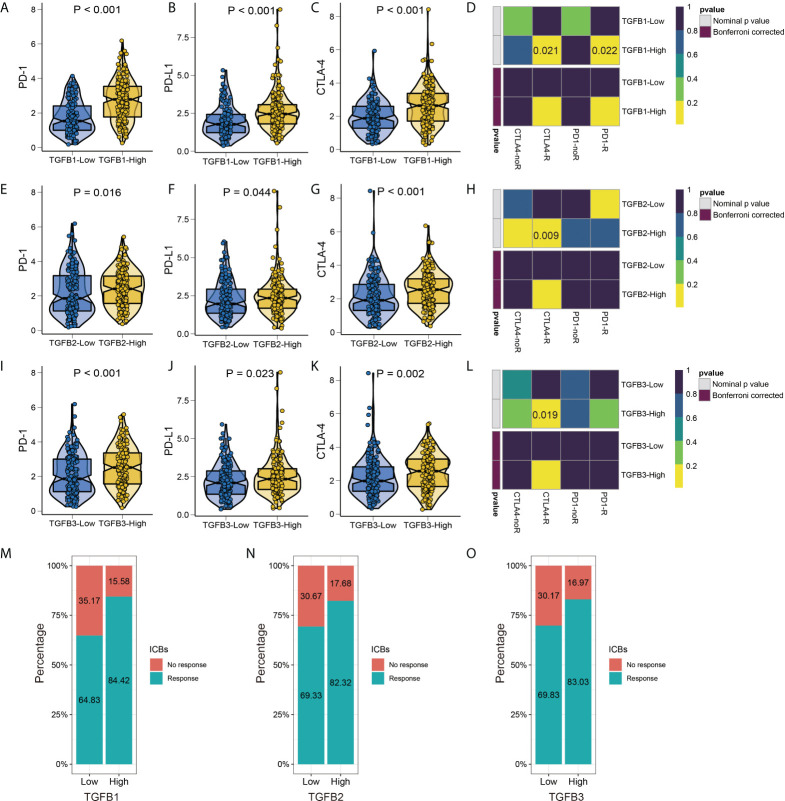
TGFβ and immunotherapy. Differences in PD–1, PD–L1 and CTLA–4 immune checkpoints between different TGFβ1 **(A–C)**, TGFβ2 **(E–G)** and TGFβ3 **(I–K)** groups. Heatmap visualized the response to anti–CTLA4 and anti–PD1 therapies between different TGFβ1 **(D)**, TGFβ2 **(H)** and TGFβ3 **(L)** groups. **(M–O)** The histogram showed the responsiveness of immunotherapy between high and low TGFβ groups, the height of each bar represents the frequency of change.

Since TGFβ is involved in several aspects of the tumor development process, we further analyzed the relationship between TGFβ and immunotherapy for gastric cancer. TGFβ was significantly positively correlated with immune checkpoint and MHC molecules in the STAD dataset (**Supplementary Figure 3A**), and PD-1, PD-L1 and CTLA-4 expression levels were higher in the high TGFβ subgroup (**Figures 10A–C, E–G, I–K**). We used two methods to assess immunotherapy response. First, the submap module results showed a response to CTLA-4 immunotherapy in the high TGFβ1, TGFβ2 and TGFβ3 subgroups, and a response to PD-1 immunotherapy in the high TGFβ1 subgroup (**Figures 10D, H, L**). In addition, using the ImmuCellAI database to further assess the guiding significance of TGFβ for ICB therapy, with high TGFβ1 (χ2 = 17.739, *P* < 0.001), TGFβ2 (χ2 = 7.978, *P* = 0.005) and TGFβ3 (χ2 = 8.237, *P* = 0.004) subgroups had a higher proportion of patients responding to immunotherapy (**Figures 10M–O**). However, within multiple gastric cancer datasets including GSE184336, the correlation of TGFβ with immune checkpoint and MHC molecules differed (**Supplementary Figure 3A**), but TGFβ still had a guiding effect on immunotherapy response (**Supplementary Figures 3B–D**). Therefore, TGFβ still needs to be analyzed and validated in more gastric cancer data sets before it can be used as a biomarker for predicting responsiveness.

### TGFβ guidance for chemotherapy

**Figure 11:**
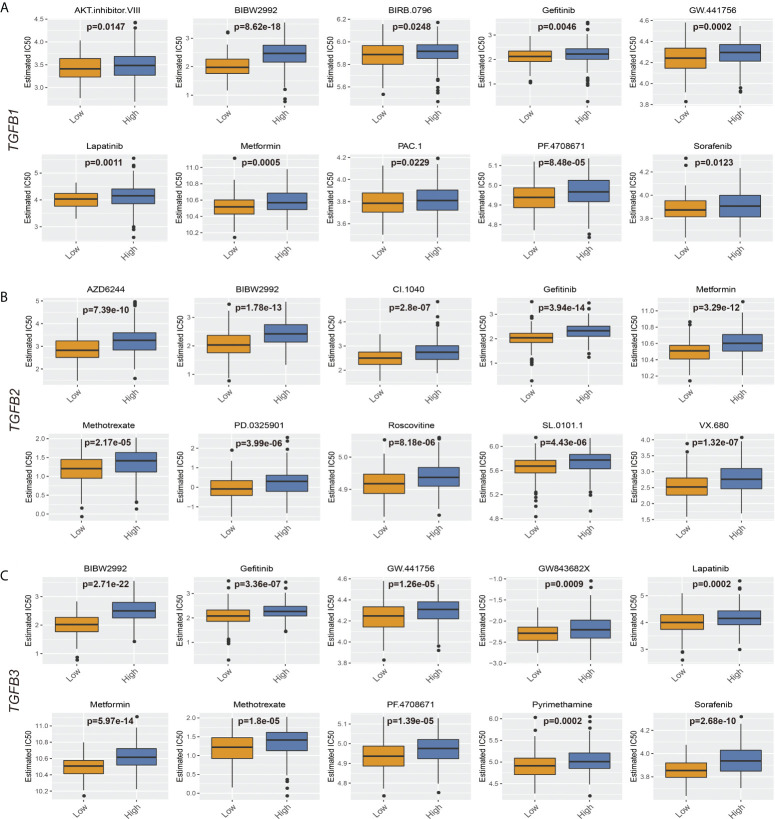
TGFβ and chemotherapy in STAD. **(A)** Boxplots depicted the differences in the estimated IC50 levels of AKT.inhibitor.VIII, BIBW2992, BIRB.0796, Gefitinib, GW.441756, Lapatinib, Metformin, PAC.1, PF.4708671 and Sorafenib between the high and low TGFβ1 groups. **(B)** Boxplots depicted the differences in the estimated IC50 levels of AZD6244, BIBW2992, CI.1040, Gefitinib, Metformin, Methotrexate, PD.0325901, Roscovitine, SL.0101.1 and VX.680 between the high and low TGFβ2 groups. **(C)** Boxplots depicted the differences in the estimated IC50 levels of BIBW2992, Gefitinib, GW.441756, GW843682X, Lapatinib, Metformin, Methotrexate, PF.4708671, Pyrimethamine and Sorafenib between the high and low TGFβ3 groups.

As an important method of adjuvant treatment for gastric cancer, chemotherapy drug therapy occupies an important position in clinical treatment. Prediction of differences in chemical drug IC50 between different TGFβ subgroups according to the GDSC database showed increased IC50 for most chemicals in the high TGFβ1, TGFβ2 and TGFβ3 expression subgroups of the STAD and GSE184336 datasets (**Figure 11**; **Supplementary Figure 4**).

## Discussion

In this study, we analyzed the transcriptomic dataset GSE184336 of 231 gastric cancer patients, and the results showed that TGFβ1, TGFβ2 and TGFβ3 were highly expressed in cancer tissues, and western blot assays further confirmed the differences in TGFβ expression. Pathological factor analysis between different TGFβ groupings in STAD and GSE184336 showed that TGFβ was associated with poorer pathological staging or grading, suggesting that TGFβ may be an important factor in the poor progression of gastric cancer. Survival analysis of GSE184336 showed shorter survival times in patients with high TGFβ1 and TGFβ2 expression, and similar results were seen in several gastric cancer datasets set. Colorectal cancer patients have high TGFβ1 levels compared to healthy controls, and high levels of TGFβ1 are positively correlated with advanced tumor stage and metastasis after surgical resection (33, 34). In addition, high levels of TGFβ2 were a factor of poor prognosis in patients with gastric cancer, which is consistent with the results reported in previous studies (35, 36). TGFβ3 was also a poor prognostic factor for STAD in this study, however, compared to the large amount of data available for TGFβ1, there is a lack of relevant data demonstrating the pathogenic role of TGFβ3 in tumorigenesis, so this study is an important addition to the role of TGFβ3 molecules in gastric cancer.

Reviewing the role and expression of TGFβ in different types of cancer, we found that TGFβ is highly expressed in most types of cancer and is one of the risk factors affecting cancer prognosis, however the mechanism by which TGFβ affects gastric cancer progression remains to be clearly defined (37–39). In the GSE184336 dataset, GSVA analysis of patients with different TGFβ1, TGFβ2 and TGFβ3 subgroups showed that high TGFβ expression significantly inhibited the TCA cycle, which plays a key role in energy metabolism and is closely related to the tissue oxygen environment. Therefore, we further analyzed the relationship between TGFβ and Hallmarks hypoxia, and the results showed that TGFβ1, TGFβ2 and TGFβ3 were significantly and positively correlated with Hallmarks hypoxia, reflecting a hypoxic microenvironment with high TGFβ factors, meanwhile the hypoxia-related genes HIF gene family and pro-angiogenic genes were highly expressed in the TGFβ high expression group. Hypoxia-inducible factor (HIF) is the main transcriptional regulator in response to hypoxia and consists of HIF-α subunits (HIF-1α or HIF-2α) and HIF-1β under hypoxic conditions (40). In earlier studies, HIF-1α was associated with TGFβ activation in hepatocytes and human umbilical vein endothelial cells during hepatic fibrosis, and TGFβ also inhibited mRNA and protein expression of PHD2, thereby increasing the stability of HIF-1α (41, 42). Overexpression of HIF-1α in breast cancer promotes the expression of TGFβ1 and SMAD3 (43). Endothelial cells in hypoxia [1% partial pressure of oxygen (PaO2)] increase messenger RNA and protein levels of TGFβ2, as well as messenger RNA levels of type II membrane receptors for TGF-beta2 (44). Therefore, the involvement of TGFβ signaling pathway in the regulation of hypoxia may be one of the important factors promoting tumor progression.

EMT is the process by which polar epithelial cells convert to migratory mesenchymal cells and gain the ability to invade and migrate, and it is present in several physiological and pathological processes in the human body (45, 46). As cancer cells diminish their epithelial characteristics during EMT, they may express fewer tumor-specific neoantigens to avoid recognition by immune cells, all of which contribute to cancer progression, with TGFβ being a key factor in EMT regulation (47, 48). The results of this study showed that TGFβ1, TGFβ2 and TGFβ3 were positively correlated with EMT, CDH2, VIM and ZEB1 and significantly negatively correlated with CDH1 in multiple gastric cancer datasets. To further verify the regulatory role of TGFβ in gastric cancer cells, we cultured gastric cancer cells with conditioned medium incorporating TGFβ1, TGFβ2 and TGFβ3 active proteins and showed that the EMT trend of cancer cells cultured under TGFβ conditions was significantly increased (CDH1 expression was decreased, CDH2, VIM and ZEB1 expression was increased), while inhibition of gastric cancer cells TGFβ1, TGFβ2 and TGFβ3 gene expression suppressed the EMT trend in gastric cancer cells (no significant change in CDH1 expression and decreased expression of CDH2 and ZEB1). It further demonstrated the important role of TGFβ gene family on the regulation of EMT.

In the gastric cancer microenvironment, TGFβ was significantly positively correlated with stromalscore and immunescore, and negatively correlated with tumor purity, and similar results were well reflected in multiple gastric cancer data sets. Another important aspect in TME was to explore the relationship between TGFβ and immune infiltration level of gastric cancer. The results showed that in gastric cancer TGFβ1, TGFβ2 and TGFβ3 expressions were strongly correlated with most of the immune cell infiltration levels. The relationship between TGFβ and immune cell infiltration was also shown for gene mutations, and somatic copy number alterations (arm-level deletion and arm-level gain) of TGFβ gene had a greater impact on immune cell infiltration. In the present study TGFβ was significantly and positively correlated with Tregs, which promote the formation of an immunosuppressive microenvironment and attenuate the antitumor effects produced by CD4+ T cells, CD8+ T cells and NK cells through secreted TGFβ (49, 50). It has been demonstrated that a decrease in the number of Tregs in different mouse models significantly increased the antitumor immune effect in mice (51). Dysfunction of anti-tumor immune cells in tumor patients is also closely related to Tregs, with a significant increase in the number of Tregs within and at the margins of tumor tissues such as gastric cancer, breast cancer, and melanoma (52–54). In TME, although the level of infiltration of immune cells with anti-tumor capacity increases with increasing levels of TGFβ expression, it is usually accompanied by a compensatory increase in immunosuppressive cells.

The results of this study showed that patients in the low TGFβ expression group had higher TMB, and patients with higher TMB had longer survival. Recent studies have shown that high TMB increases the likelihood that immunogenic neoantigens expressed by tumor cells induce a response to immunotherapy. In addition, TGFβ1 and TGFβ3 were negatively associated with MATH, which is prevalent in most cancer patients and is a major driver of acquired resistance to cancer therapy (55, 56). In the context of immunotherapy, the pressure on the immune system to respond to specific tumor antigens can drive selection against antigen-negative cells, which is a common cause of clinical relapse. In the present study MATH was lower when TGFβ1 and TGFβ3 were higher, while higher levels of TGFβ promoted the formation of an immunosuppressive microenvironment and facilitated the progression of EMT, conditions that favored the evolutionary development of cancer cells.

Stromal fibroblasts and other cells in tumor tissues shape the immunosuppressive environment of tumors through TGFβ signaling, inhibiting the antitumor activity of immune cells and preventing or weakening the effect of anticancer immunotherapy (57). Therefore, inhibition of TGFβ signaling is considered as a prerequisite and an important way to improve the effectiveness of immunotherapy. Considering TGFβ, CTLA4 and PD-L1/PD-1 as parallel immunosuppressive pathways, combining TGFβ inhibitors with other immune checkpoint inhibitors may improve the therapeutic efficacy (58–61). The results of this study showed that the expression levels of immune checkpoints PD-1, PD-L1 and CTLA-4 were higher in the high TGFβ subgroup, and in addition we predicted the response of TGFβ expression levels to ICB therapy, with a higher proportion of patients in the high TGFβ subgroup responding to ICB therapy. Combination therapy has been pre-evaluated in mouse cancer models where, according to the model and experimental design, therapeutic co-administration of TGFβ blockade and anti-PD-L1 antibodies reduced TGFβ signaling in stromal cells, promoted T-cell infiltration into tumor centers, and provoked potent antitumor immunity and tumor regression (62).

Furthermore, in drug prediction of patients with different TGFβ subgroups, it was found that patients in the high TGFβ group were less sensitive to treatment with small molecule compounds, which may be related to the increased extracellular interstitial component of gastric cancer induced by high TGFβ levels. The rigid Extracellular matrix (ECM) that forms around the tumor reduces the spread of therapeutic agents to cancer cells, while the dense ECM reduces the vascular density and causes the vessels to embed in the matrix, forming a tough barrier that cannot be perfused with drugs (63).

However there are many clinical challenges in developing TGFβ inhibitors, especially patient selection and timing of treatment. Considering the dual role of TGFβ on proliferation, TGFβ inhibitors may be beneficial in advanced tumors. It is worth noting that TGFβ plays a positive factor in SKCM, KIRC and THYM in pan-cancer analysis and should be considered carefully when using TGFβ-related signaling inhibitors. Before TGFβ inhibitors can be used clinically, a lot of research on different types of cancer is still needed. To comprehensively evaluate the efficacy of TGFβ inhibitors in immunotherapy in different cancer types and cancer stages, and whether they need to be used in combination with other immune target inhibitors. Only then can we make accurate screening and evaluation of the target population and therapeutic efficacy of TGFβ inhibitors.

## Data availability statement

The datasets presented in this study can be found in online repositories. The names of the repository/repositories and accession number(s) can be found in the article/**Supplementary Material**.

## Ethics statement

The studies involving human participants were reviewed and approved by Research Ethics Committee of the Affiliated Cancer Hospital of Harbin Medical University. The patients/participants provided their written informed consent to participate in this study.

## Author contributions

BH and TF conceived the project and wrote the manuscript. BH and YZ participated in data analysis. JG and YLZ participated in discussion and language editing. YX reviewed the manuscript. All authors contributed to the article and approved the submitted version.

## Funding

This study was supported by the Harbin Science and Technology Bureau Research and Development Project of Applied Technology (No. 2017RAXXJ054) and Nn 10 Program of Harbin Medical University Cancer Hospital (No. Nn 10 PY 2017-03).

## Acknowledgments

We sincerely thank all participants in the study.

## Conflict of interest

The authors declare that the research was conducted in the absence of any commercial or financial relationships that could be construed as a potential conflict of interest.

## Publisher’s note

All claims expressed in this article are solely those of the authors and do not necessarily represent those of their affiliated organizations, or those of the publisher, the editors and the reviewers. Any product that may be evaluated in this article, or claim that may be made by its manufacturer, is not guaranteed or endorsed by the publisher.

## Supplementary material

Supplementary Figure 1 TGFβ network analysis. **(A)** Using GeneMANIA to study functional associations between different proteins, the colors of the connections represent different correlations, and the different colors in the circles represent the different functions involved. **(B)** Regulation of TGFβ signaling pathway.

Supplementary Figure 2 Analysis of copy number variation among different TGFβ groups. **(A, C, E)** Comparison of the frequency of copy number changes in different TGFβ groupings. Chromosomal locations of peaks of significantly recurring focal amplification (red) and deletions (blue) were presented. **(B–D)** Comparison of tumor infiltration levels between tumors with different somatic copy number alterations of TGFβ. * *P* < 0.05; ** *P* < 0.01; ****P* < 0.001.

Supplementary Figure 3 TGFβ with MHC molecules and Immune checkpoints. **(A)** Correlation of TGFβ with MHC molecules and Immune checkpoints. **(B–D)** The expression levels of TGFβ1, TGFβ2 and TGFβ3 in different immunotherapy response subgroups. * *P* < 0.05; ** *P* < 0.01; *** *P* < 0.001; **** *P* < 0.0001.

Supplementary Figure 4 TGFβ and chemotherapy in GSE184336. **(A)** Boxplots depicted the differences in the estimated IC50 levels of AKT.inhibitor.VIII, BIBW2992, BMS.708163, Gefitinib, GW.441756, Lapatinib, Metformin, PF.4708671, Roscovitine and Sorafenib between the high and low TGFβ1 groups. **(B)** Boxplots depicted the differences in the estimated IC50 levels of AKT.inhibitor.VIII, AZD6244, BIBW2992, Erlotinib, Gefitinib, GW.441756, Lapatinib, Metformin, Roscovitine and Sorafenib between the high and low TGFβ2 groups. **(C)** Boxplots depicted the differences in the estimated IC50 levels of A.443654, AKT.inhibitor.VIII, BIBW2992, Gefitinib, Lapatinib, Metformin, Paclitaxel, PF.4708671, Roscovitine and Sorafenib between the high and low TGFβ3 groups.
