# Supplementary figures and images for: Association of the TGFβ gene family with microenvironmental features of gastric cancer and prediction of response to immunotherapy
Source: Front Oncol. 2022 Sep 2;12:920599. doi: 10.3389/fonc.2022.920599 (PMC9478444; doi:10.3389/fonc.2022.920599)

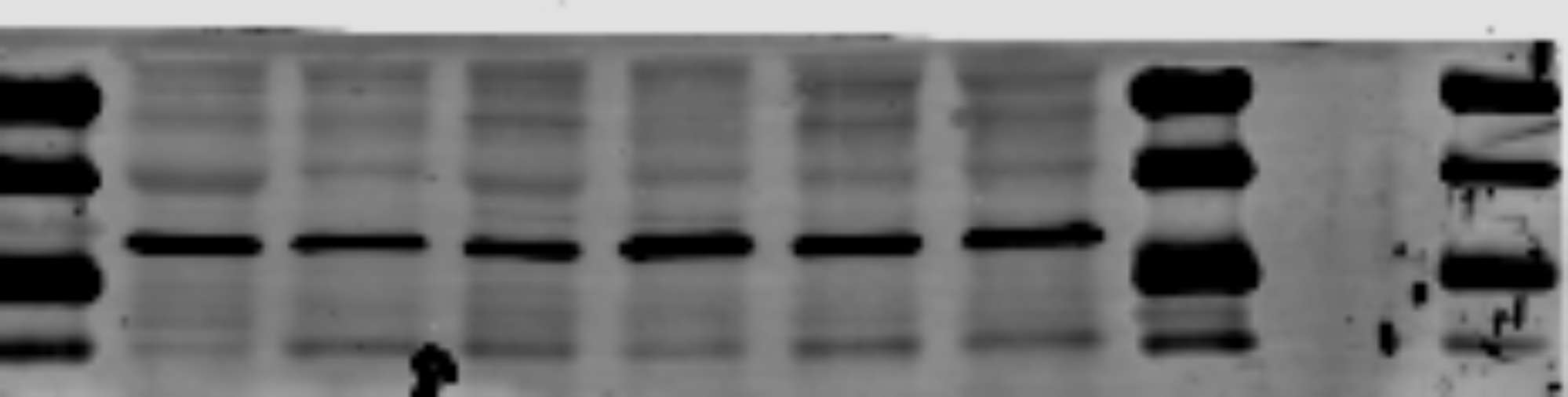

Supplement: Supplementary file 1 [file DataSheet_1.zip › Western blot/GAPDH_1.tif]

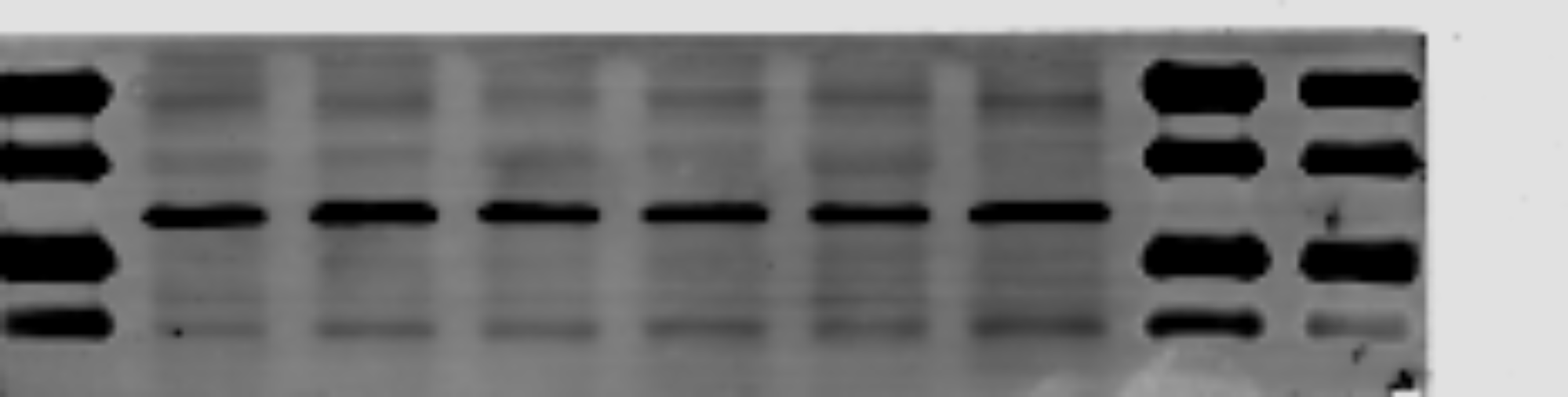

Supplement: Supplementary file 1 [file DataSheet_1.zip › Western blot/GAPDH_2.tif]

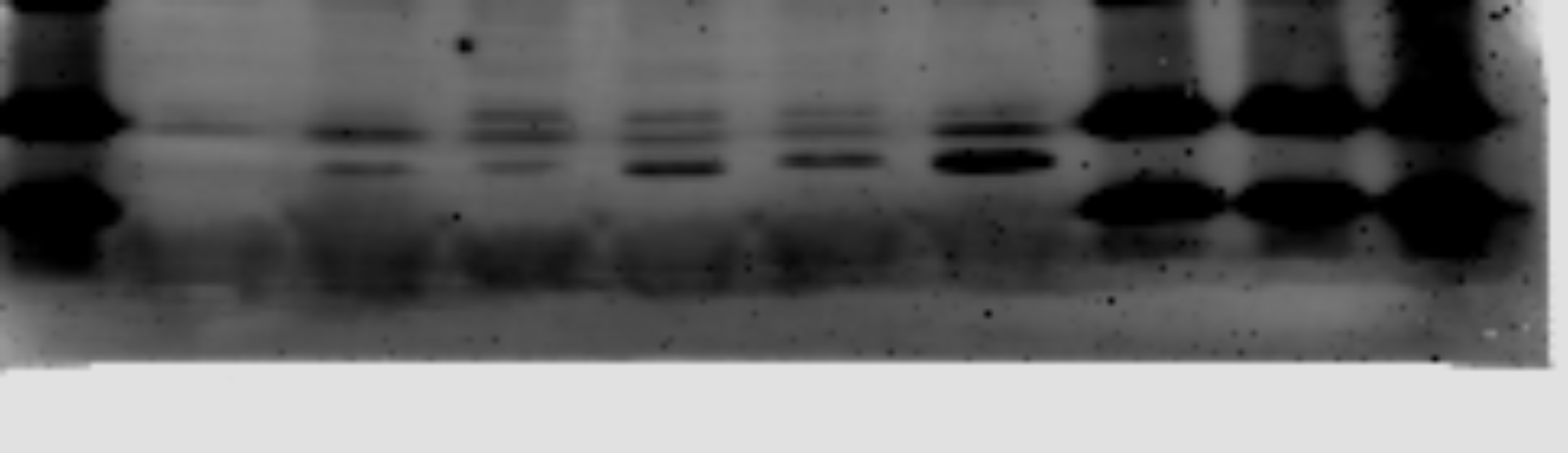

Supplement: Supplementary file 1 [file DataSheet_1.zip › Western blot/TGFB1_1.tif]

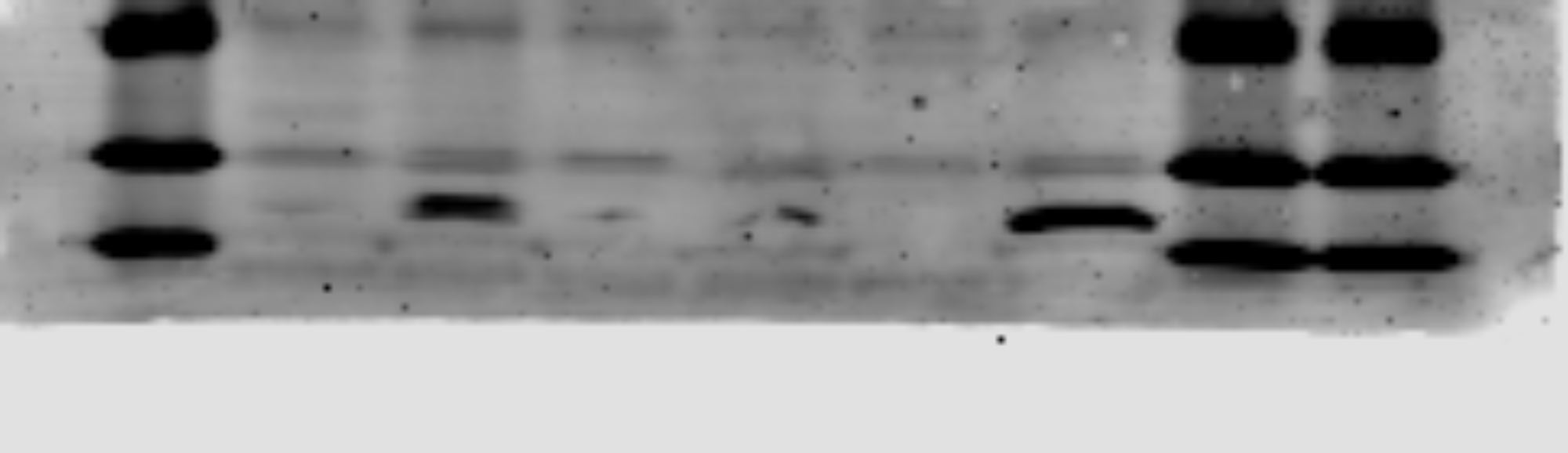

Supplement: Supplementary file 1 [file DataSheet_1.zip › Western blot/TGFB1_2.tif]

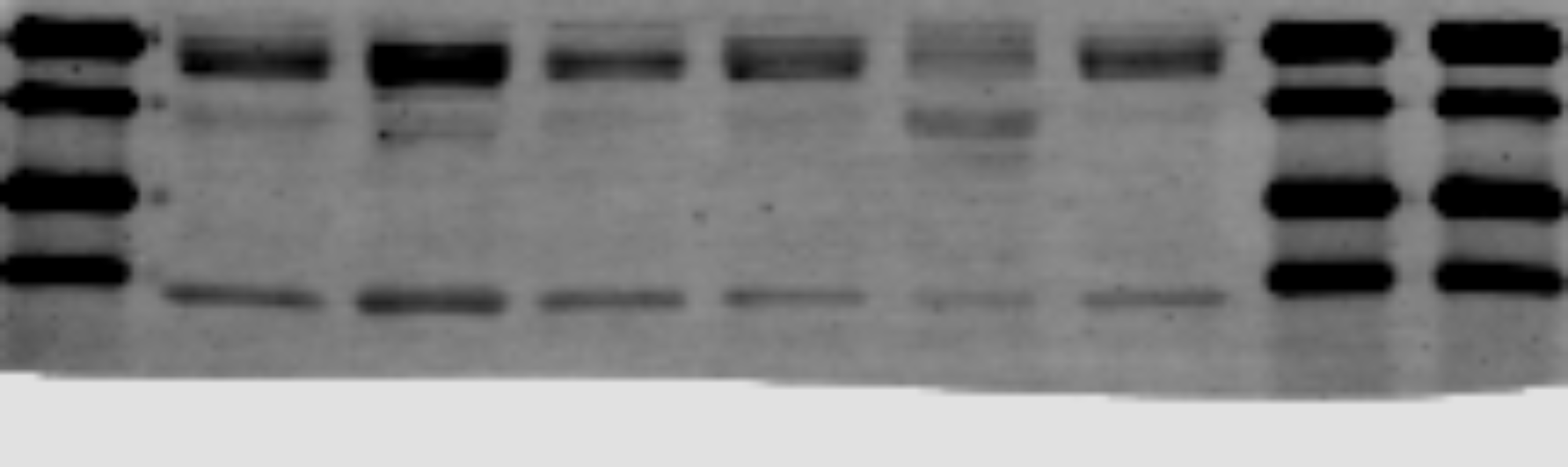

Supplement: Supplementary file 1 [file DataSheet_1.zip › Western blot/TGFB2_1.tif]

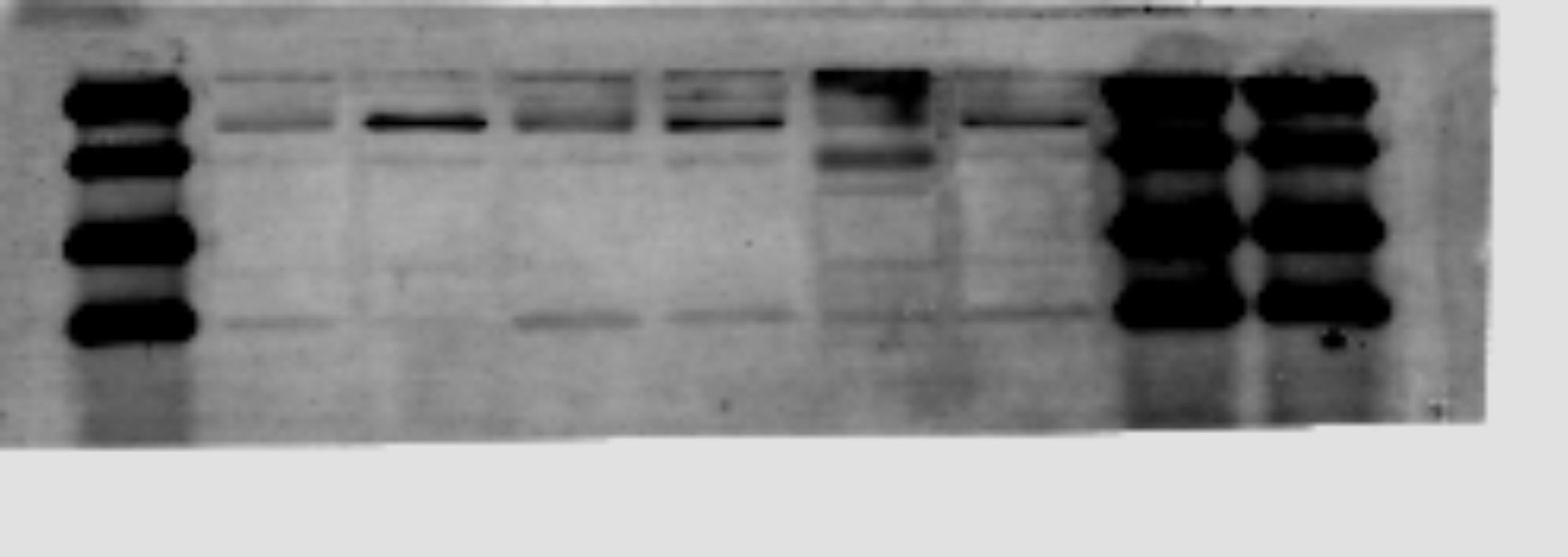

Supplement: Supplementary file 1 [file DataSheet_1.zip › Western blot/TGFB2_2.tif]

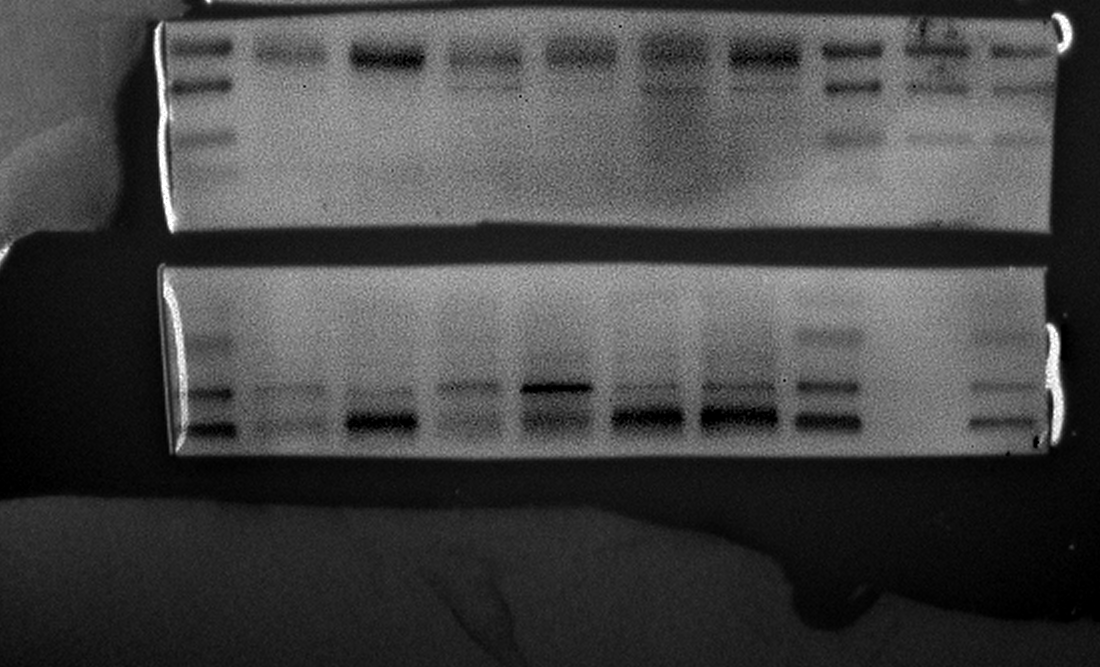

Supplement: Supplementary file 1 [file DataSheet_1.zip › Western blot/TGFB3.png]

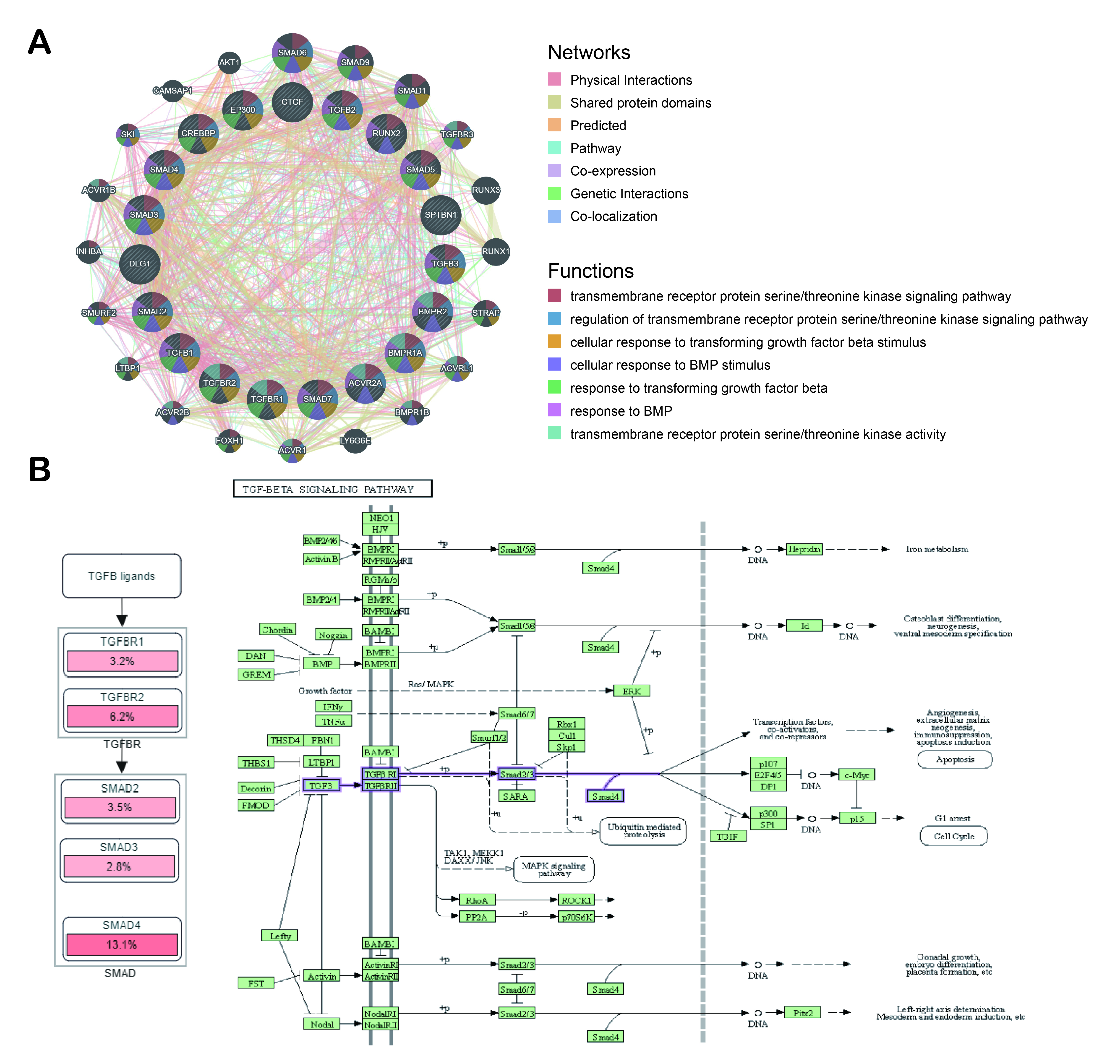

Supplement: Supplementary Figure 1 — TGFβ network analysis. (A) Using GeneMANIA to study functional associations between different proteins, the colors of the connections represent different correlations, and the different colors in the circles represent the different functions involved. (B) Regulation of TGFβ signaling pathway. [file Image_1.jpeg]

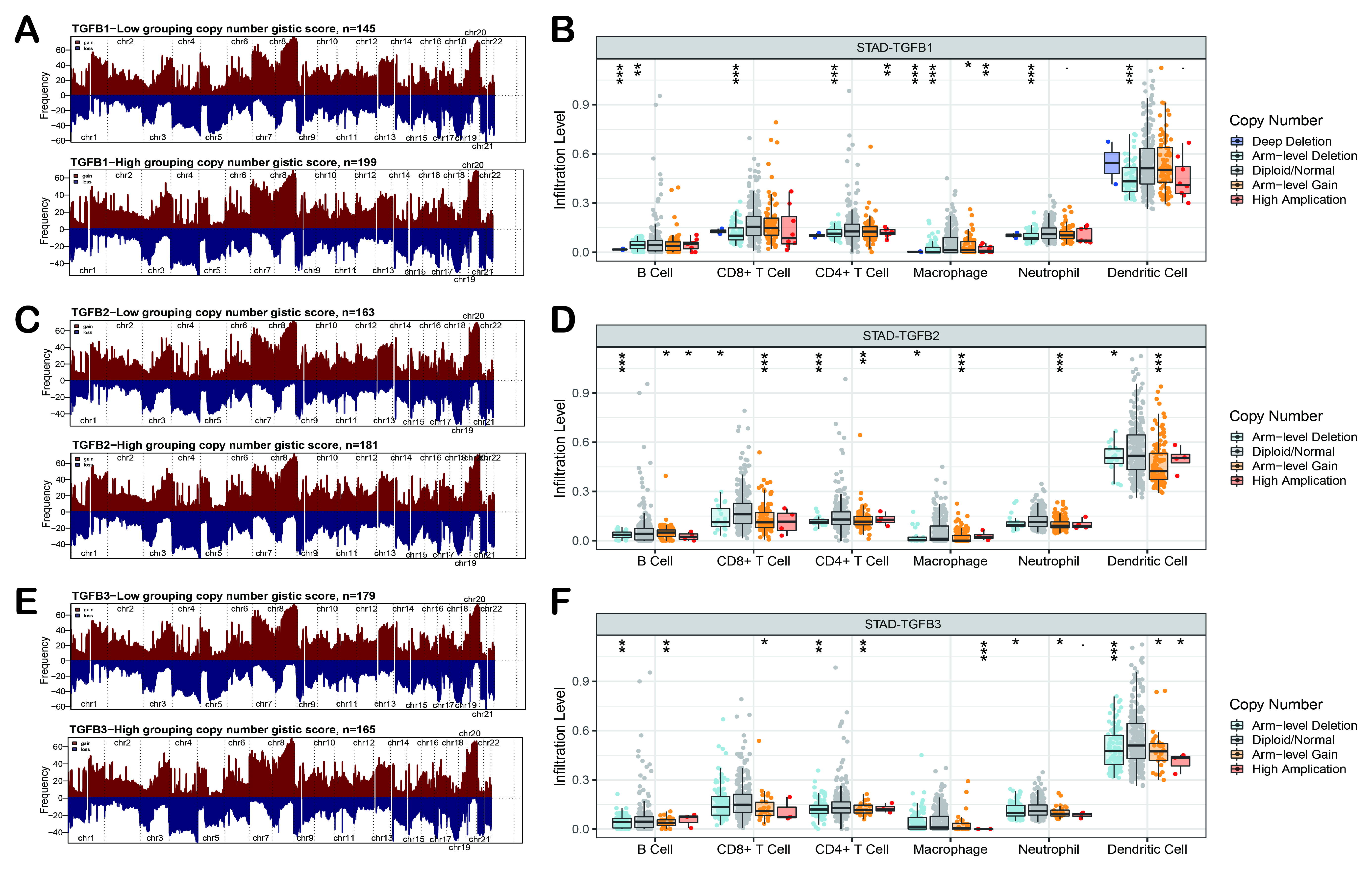

Supplement: Supplementary Figure 2 — Analysis of copy number variation among different TGFβ groups. (A, C, E) Comparison of the frequency of copy number changes in different TGFβ groupings. Chromosomal locations of peaks of significantly recurring focal amplification (red) and deletions (blue) were presented. (B–D) Comparison of tumor infiltration levels between tumors with different somatic copy number alterations of TGFβ. * P < 0.05; ** P < 0.01; ***P < 0.001. [file Image_2.jpeg]

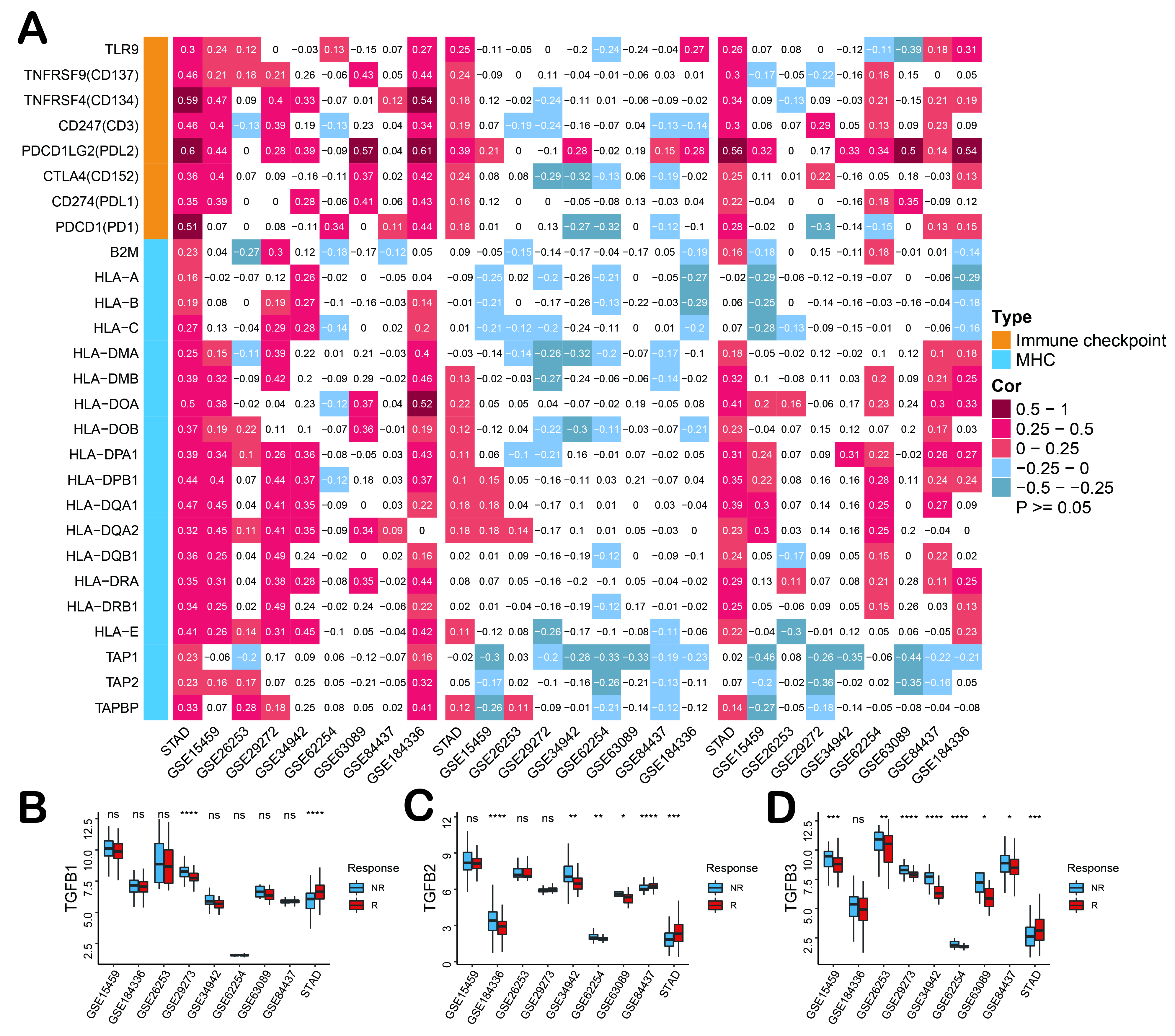

Supplement: Supplementary Figure 3 — TGFβ with MHC molecules and Immune checkpoints. (A) Correlation of TGFβ with MHC molecules and Immune checkpoints. (B–D) The expression levels of TGFβ1, TGFβ2 and TGFβ3 in different immunotherapy response subgroups. * P < 0.05; ** P < 0.01; *** P < 0.001; **** P < 0.0001. [file Image_3.jpeg]

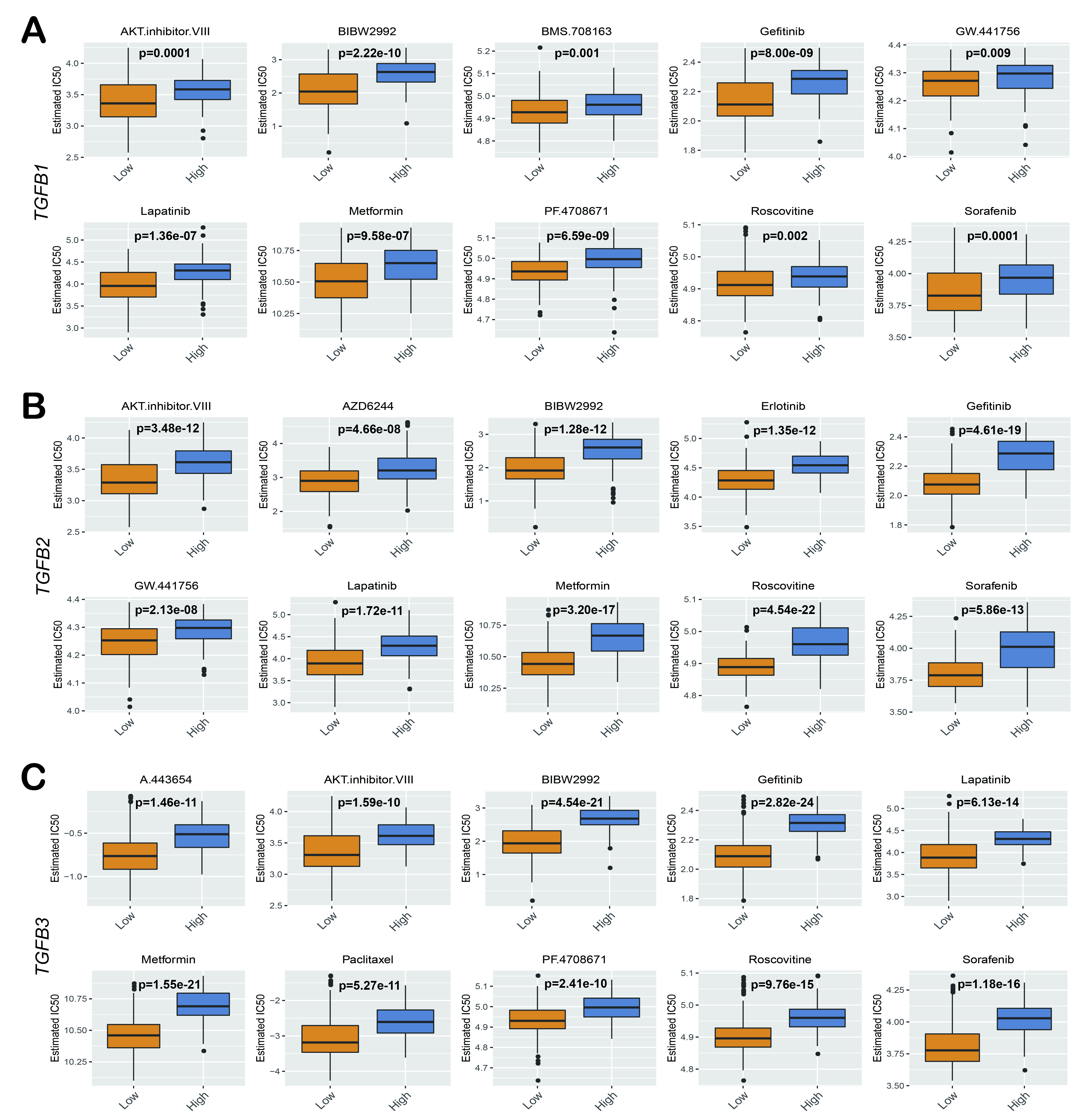

Supplement: Supplementary Figure 4 — TGFβ and chemotherapy in GSE184336. (A) Boxplots depicted the differences in the estimated IC50 levels of AKT.inhibitor.VIII, BIBW2992, BMS.708163, Gefitinib, GW.441756, Lapatinib, Metformin, PF.4708671, Roscovitine and Sorafenib between the high and low TGFβ1 groups. (B) Boxplots depicted the differences in the estimated IC50 levels of AKT.inhibitor.VIII, AZD6244, BIBW2992, Erlotinib, Gefitinib, GW.441756, Lapatinib, Metformin, Roscovitine and Sorafenib between the high and low TGFβ2 groups. (C) Boxplots depicted the differences in the estimated IC50 levels of A.443654, AKT.inhibitor.VIII, BIBW2992, Gefitinib, Lapatinib, Metformin, Paclitaxel, PF.4708671, Roscovitine and Sorafenib between the high and low TGFβ3 groups. [file Image_4.jpeg]
