# Supplementary material for: Association of the TGFβ gene family with microenvironmental features of gastric cancer and prediction of response to immunotherapy
Source: Front Oncol. 2022 Sep 2;12:920599. doi: 10.3389/fonc.2022.920599 (PMC9478444; doi:10.3389/fonc.2022.920599)
Supplement: Supplementary file 7 [file Table_2.docx]

**Supplementary TABLE 2 |** siRNA sequence.

**siRNA-TGFβ1**

| siRNA | Sequence (5’-3’) |
| --- | --- |
| TGFβ1(human)siRNA 1-1 | ACAACGAAAUCUAUGACAATT |
|  | UUGUCAUAGAUUUCGUUGUTT |
| TGFβ1(human)siRNA 1-2 | CAGAAAUACAGCAACAAUUTT |
|  | AAUUGUUGCUGUAUUUCUGTT |
| TGFβ1(human)siRNA 1-3 | GGACUAUCCACCUGCAAGATT |
|  | UCUUGCAGGUGGAUAGUCCTT |

**siRNA-TGFβ2**

| siRNA | Sequence (5’-3’) |
| --- | --- |
| TGFβ2(human)siRNA 2-1 | CGACAGCAAAGUUGUGAAATT |
|  | UUUCACAACUUUGCUGUCGTT |
| TGFβ2(human)siRNA 2-2 | CCAAGGAGGUUUACAAAAUTT |
|  | AUUUUGUAAACCUCCUUGGTT |
| TGFβ2(human)siRNA 2-3 | CUAUAAAGUCCACUAGGAATT |
|  | UUCCUAGUGGACUUUAUAGTT |

**siRNA-TGFβ3**

| siRNA | Sequence (5’-3’) |
| --- | --- |
| TGFβ3(human)siRNA 3-1 | GGGGACAGAUCUUGAGCAATT |
|  | UUGCUCAAGAUCUGUCCCCTT |
| TGFβ3(human)siRNA 3-2 | CAACCUAUUCCGAGCAGAATT |
|  | UUCUGCUCGGAAUAGGUUGTT |
| TGFβ3(human)siRNA 3-3 | GCGUGGACAAUGAGGAUGATT |
|  | UCAUCCUCAUUGUCCACGCTT |

**siRNA-NC**

| Primer | Primer sequence (5’-3’) |
| --- | --- |
| siRNA-NC | UUCUCCGAACGUGUCACGUTT |
|  | ACGUGACACGUUCGGAGAATT |
