# Supplementary material for: Association of the TGFβ gene family with microenvironmental features of gastric cancer and prediction of response to immunotherapy
Source: Front Oncol. 2022 Sep 2;12:920599. doi: 10.3389/fonc.2022.920599 (PMC9478444; doi:10.3389/fonc.2022.920599)
Supplement: Supplementary file 8 [file Table_3.docx]

**Supplementary TABLE 3 |** Primer sequence.

| Genes | Gene ID | Primer sequence (5’-3’) |
| --- | --- | --- |
| TGFβ1 | 7040 | For: GGCCAGATCCTGTCCAAGC |
|  |  | Rev: GTGGGTTTCCACCATTAGCAC |
| TGFβ2 | 7042 | For: CAGCACACTCGATATGGACCA |
|  |  | Rev:CCTCGGGCTCAGGATAGTCT |
| TGFβ3 | 7043 | For: ACTTGCACCACCTTGGACTTC |
|  |  | Rev: GGTCATCACCGTTGGCTCA |
| CDH1 | 999 | For: ATTTTTCCCTCGACACCCGAT |
|  |  | Rev: TCCCAGGCGTAGACCAAGA |
| CDH2 | 1000 | For: TCAGGCGTCTGTAGAGGCTT |
|  |  | Rev:ATGCACATCCTTCGATAAGACTG |
| VIM | 7431 | For:GACGCCATCAACACCGAGTT |
|  |  | Rev:CTTTGTCGTTGGTTAGCTGGT |
| ZEB1 | 6935 | For:GATGATGAATGCGAGTCAGATGC |
|  |  | Rev:ACAGCAGTGTCTTGTTGTTGT |
| β-actin | 60 | For: TGACGTGGACATCCGCAAAG |
|  |  | Rev: CTGGAAGGTGGACAGCGAGG |
