# Supplementary material for: Association of the TGFβ gene family with microenvironmental features of gastric cancer and prediction of response to immunotherapy
Source: Front Oncol. 2022 Sep 2;12:920599. doi: 10.3389/fonc.2022.920599 (PMC9478444; doi:10.3389/fonc.2022.920599)
Supplement: Supplementary file 9 [file Table_4.docx]

**Supplementary TABLE 4 |** The relationship between TGFβ1 expression and clinicopathological factors in HMUCH (GSE184336) and TCGA-STAD database.

| Clinical features | Total | TGFβ1 expression (HMUCH) | | *P*-value |  | Total | TGFβ1 expression (STAD) | | *P*-value |
| --- | --- | --- | --- | --- | --- | --- | --- | --- | --- |
|  |  | Low (%) | High (%) |  |  |  | Low (%) | High (%) |  |
| Age  <60  ≥60 | 114  117 | 76(50.0%)  76(50.0%) | 38(48.1%)  41(51.9%) | 0.784 |  | 105  236 | 46(31.9%)  98(68.1%) | 59(29.9%)  138(70.1%) | 0.693 |
| Gender  Female  Male | 83  148 | 52(34.2%)  100(65.8%) | 31(39.2%)  48(60.8%) | 0.450 |  | 122  222 | 54(37.2%)  91(62.8%) | 68(34.2%)  131(65.8%) | 0.557 |
| TNM stage  Ⅰ  Ⅱ  Ⅲ  Ⅳ | 36  49  129  17 | 31(20.4%)  36(23.7%)  75(49.3%)  10(6.6%) | 5(6.2%)  13(16.5%)  54(68.4%)  7(8.9%) | **0.009** |  | 47  107  144  38 | 25(17.4%)  44(30.6%)  59(41.0%)  16(11.1%) | 22(11.5%)  63(32.8%)  85(44.3%)  22(11.5%) | 0.494 |
| T stage  T1  T2  T3  T4 | 21  25  142  43 | 17(11.2%)  22(14.5%)  89(58.6%)  24(15.8%) | 4(5.1%)  3(3.8%)  53(67.1%)  19(24.1%) | **0.017** |  | 17  74  160  69 | 14(9.7%)  27(18.6%)  70(48.3%)  34(23.4%) | 3(1.5%)  47(24.1%)  90(46.2%)  55(28.2%) | **0.005** |
| N stage  N0  N1  N2  N3 | 65  29  44  93 | 49(32.2%)  25(16.4%)  28(18.4%)  50(32.9%) | 16(20.3%)  4(5.1%)  16(20.3%)  43(54.4%) | **0.003** |  | 99  94  71  70 | 46(31.9%)  39(27.1%)  33(22.9%)  26(18.1%) | 53(27.9%)  55(28.9%)  38(20.0%)  44(23.2%) | 0.593 |
| Histologic Grade  G1  G2  G3 | 4  92  135 | 3(2.0%)  64(42.1%)  85(55.9%) | 1(1.3%)  28(35.4%)  50(63.3%) | 0.545 |  | 9  124  202 | 4(2.8%)  64(45.4%)  73(51.8%) | 5(2.6%)  60(30.9%)  129(66.5%) | **0.023** |

Bold values indicate *P-value* < 0.05.
