# Supplementary material for: Association of the TGFβ gene family with microenvironmental features of gastric cancer and prediction of response to immunotherapy
Source: Front Oncol. 2022 Sep 2;12:920599. doi: 10.3389/fonc.2022.920599 (PMC9478444; doi:10.3389/fonc.2022.920599)
Supplement: Supplementary file 10 [file Table_5.docx]

**Supplementary TABLE 5 |** The relationship between TGFβ2 expression and clinicopathological factors in HMUCH (GSE184336) and TCGA database.

| Clinical features | Total | TGFβ2 expression (HMUCH) | | *P*-value |  | Total | TGFβ2 expression (STAD) | | *P*-value |
| --- | --- | --- | --- | --- | --- | --- | --- | --- | --- |
|  |  | Low (%) | High (%) |  |  |  | Low (%) | High (%) |  |
| Age  <60  ≥60 | 114  117 | 56(50.0%)  56(50.0%) | 58(48.7%)  61(51.3%) | 0.848 |  | 105  236 | 50(30.7%)  113(69.3%) | 55(30.9%)  123(69.1%) | 0.964 |
| Gender  Female  Male | 83  148 | 38(33.9%)  74(66.1%) | 45(37.8%)  74(62.2%) | 0.538 |  | 122  222 | 62(38.0%)  101(62.0%) | 60(33.1%)  121(66.9%) | 0.344 |
| TNM stage  Ⅰ  Ⅱ  Ⅲ  Ⅳ | 36  49  129  17 | 20(17.9%)  27(24.1%)  57(50.9%)  8(7.1%) | 16(13.4%)  22(18.5%)  72(60.5%)  9(7.6%) | 0.467 |  | 47  107  144  38 | 27(16.9%)  51(31.9%)  64(40.0%)  18(11.3%) | 20(11.4%)  56(31.8%)  80(45.4%)  20(11.4%) | 0.493 |
| T stage  T1  T2  T3  T4 | 21  25  142  43 | 11(9.8%)  16(14.3%)  68(60.7%)  17(15.2%) | 10(8.4%)  9(7.6%)  74(62.2%)  26(21.8%) | 0.268 |  | 17  74  160  69 | 13(8.0%)  34(20.9%)  78(47.9%)  38(23.2%) | 4(2.3%)  40(22.6%)  82(46.3%)  51(28.8%) | 0.083 |
| N stage  N0  N1  N2  N3 | 65  29  44  93 | 33(29.5%)  19(17.0%)  22(19.6%)  38(33.9%) | 32(26.9%)  10(8.4%)  22(18.5%)  55(46.2%) | 0.127 |  | 99  94  71  70 | 52(32.7%)  43(27.0%)  31(19.5%)  33(20.8%) | 47(26.9%)  51(28.1%)  40(22.9%)  37(21.1%) | 0.673 |
| Histologic Grade  G1  G2  G3 | 4  92  135 | 3(2.7%)  44(39.3%)  65(58.0%) | 1(0.8%)  48(40.3%)  70(58.9%) | 0.563 |  | 9  124  202 | 6(3.7%)  68(42.0%)  88(54.3%) | 3(1.7%)  56(32.4%)  114(65.9%) | 0.076 |

Bold values indicate *P-value* < 0.05.
