# Supplementary material for: Association of the TGFβ gene family with microenvironmental features of gastric cancer and prediction of response to immunotherapy
Source: Front Oncol. 2022 Sep 2;12:920599. doi: 10.3389/fonc.2022.920599 (PMC9478444; doi:10.3389/fonc.2022.920599)
Supplement: Supplementary file 11 [file Table_6.docx]

**Supplementary TABLE 6 |** The relationship between TGFβ3 expression and clinicopathological factors in HMUCH (GSE184336) and TCGA database.

| Clinical features | Total | TGFβ3 expression (HMUCH) | | *P*-value |  | Total | TGFβ3 expression (STAD) | | *P*-value |
| --- | --- | --- | --- | --- | --- | --- | --- | --- | --- |
|  |  | Low (%) | High (%) |  |  |  | Low (%) | High (%) |  |
| Age  <60  ≥60 | 114  117 | 71(50.7%)  69(49.3%) | 43(47.3%)  48(52.7%) | 0.607 |  | 105  236 | 51(28.8%)  126(71.2%) | 54(32.9%)  110(67.1%) | 0.411 |
| Gender  Female  Male | 83  148 | 50(35.7%)  90(64.3%) | 33(36.3%)  58(63.7%) | 0.932 |  | 122  222 | 62(34.6%)  117(65.4%) | 60(36.4%)  105(63.6%) | 0.738 |
| TNM stage  Ⅰ  Ⅱ  Ⅲ  Ⅳ | 36  49  129  17 | 34(24.3%)  32(22.9%)  64(45.7%)  10(7.1%) | 2(2.2%)  17(18.7%)  65(71.4%)  7(7.7%) | **<0.001** |  | 47  107  144  38 | 34(19.2%)  51(28.8%)  74(41.8%)  18(10.2%) | 13(8.2%)  56(35.2%)  70(44.0%)  20(12.6%) | **0.031** |
| T stage  T1  T2  T3  T4 | 21  25  142  43 | 19(13.6%)  23(16.4%)  74(52.9%)  24(17.1%) | 2(2.2%)  2(2.2%)  68(74.7%)  19(20.9%) | **<0.001** |  | 17  74  160  69 | 17(9.5%)  42(23.5%)  80(44.7%)  40(22.3%) | 0(0.0%)  32(19.9%)  80(49.7%)  49(30.4%) | **<0.001** |
| N stage  N0  N1  N2  N3 | 65  29  44  93 | 54(38.6%)  19(13.6%)  24(17.1%)  43(30.7%) | 11(12.1%)  10(11.0%)  20(22.0%)  50(54.9%) | **<0.001** |  | 99  94  71  70 | 53(30.1%)  50(28.4%)  39(22.2%)  34(19.3%) | 46(29.1%)  44(27.8%)  32(20.3%)  36(22.8%) | 0.883 |
| Histologic Grade  G1  G2  G3 | 4  92  135 | 4(2.9%)  60(42.9%)  76(54.2%) | 0(0.0%)  32(35.2%)  59(64.8%) | 0.107 |  | 9  124  202 | 5(2.8%)  82(46.6%)  89(50.6%) | 4(2.5%)  42(26.4%)  113(71.1%) | **0.001** |

Bold values indicate *P-value* < 0.05.
