# Supplementary material for: Association of the TGFβ gene family with microenvironmental features of gastric cancer and prediction of response to immunotherapy
Source: Front Oncol. 2022 Sep 2;12:920599. doi: 10.3389/fonc.2022.920599 (PMC9478444; doi:10.3389/fonc.2022.920599)
Supplement: Supplementary file 12 [file Table_7.docx]

**Supplementary TABLE 7 |** The correlation between TGFβ expression and different immune cell marker genes (TIMER).

| **Description** | **Gene markers** | **STAD-TGFβ1** | | | | | | |  | | **STAD-TGFβ2** | | | | | |  | | **STAD-TGFβ3** | | | | | |
| --- | --- | --- | --- | --- | --- | --- | --- | --- | --- | --- | --- | --- | --- | --- | --- | --- | --- | --- | --- | --- | --- | --- | --- | --- |
|  |  | **None** | |  | | **Purity** | | |  | | **None** | |  | | **Purity** | |  | | **None** | |  | | **Purity** | |
|  |  | **Cor** | ***P*** |  | | | **Cor** | ***P*** |  | | **Cor** | ***P*** |  | | **Cor** | ***P*** |  | | **Cor** | ***P*** |  | | **Cor** | ***P*** |
| CD8+ T cell | CD8A  CD8B | 0.429  0.323 | ***  *** | | 0.413  0.314 | | | ***  *** | | 0.169  0.15 | | **  * | | 0.174  0.161 | | **  * | | 0.318  0.189 | | ***  ** | | 0.298  0.177 | | ***  ** |
| T cell (general) | CD3D  CD3E  CD2 | 0.403  0.423  0.419 | ***  ***  *** | | 0.382  0.408  0.401 | | | ***  ***  *** | | 0.082  0.109  0.16 | | 0.097  0.026  * | | 0.082  0.108  0.169 | | 0.109  0.036  ** | | 0.244  0.275  0.321 | | ***  ***  *** | | 0.214  0.248  0.301 | | ***  ***  *** |
| B cell | CD19  CD79A | 0.397  0.421 | ***  *** | | 0.375  0.386 | | | ***  *** | | 0.153  0.149 | | *  * | | 0.132  0.129 | | 0.010  0.012 | | 0.265  0.269 | | ***  *** | | 0.246  0.233 | | ***  *** |
| Monocyte | CD86  CD115 | 0.494  0.59 | ***  *** | | 0.473  0.573 | | | ***  *** | | 0.242  0.389 | | ***  *** | | 0.252  0.384 | | ***  *** | | 0.489  0.584 | | ***  *** | | 0.475  0.569 | | ***  *** |
| TAM | CCL2  CD68  IL10 | 0.521  0.361  0.486 | ***  ***  *** | | 0.498  0.34  0.478 | | | ***  ***  *** | | 0.337  0.153  0.338 | | ***  *  *** | | 0.351  0.159  0.359 | | ***  *  *** | | 0.549  0.231  0.51 | | ***  ***  *** | | 0.523  0.203  0.512 | | ***  ***  *** |
| M1 Macrophage | INOS  IRF5  COX2 | 0.277  0.453  0.117 | ***  ***  0.017 | | 0.3  0.457  0.111 | | | ***  ***  0.031 | | 0.265  0.263  0.348 | | ***  ***  *** | | 0.258  0.274  0.35 | | ***  ***  *** | | 0.261  0.289  0.205 | | ***  ***  *** | | 0.229  0.285  0.205 | | ***  ***  *** |
| M2 Macrophage | CD163  VSIG4  MS4A4A | 0.486  0.511  0.494 | ***  ***  *** | | 0.471  0.514  0.482 | | | ***  ***  *** | | 0.333  0.331  0.322 | | ***  ***  *** | | 0.334  0.359  0.334 | | ***  ***  *** | | 0.545  0.575  0.564 | | ***  ***  *** | | 0.533  0.517  0.556 | | ***  ***  *** |
| Neutrophils | CD66b  CD11b  CCR7 | –0.043  0.516  0.492 | 0.385  ***  *** | | –0.044  0.504  0.478 | | | 0.388  ***  *** | | 0.04  0.342  0.256 | | 0.413  ***  *** | | 0.05  0.352  0.26 | | 0.336  ***  *** | | -0.065  0.507  0.353 | | 0.188  ***  *** | | -0.007  0.498  0.329 | | 0.134  ***  *** |
| Natural killer cell | KIR2DL1  KIR2DL3  KIR2DL4  KIR3DL1  KIR3DL2  KIR3DL3  KIR2DS4 | 0.097  0.044  0.056  0.114  0.182  –0.001  0.059 | 0.048  0.372  0.256  0.02  **  0.9880.227 | | 0.088  0.018  0.046  0.089  0.168  0.011  0.037 | | | 0.088  0.731  0.369  0.085  *  0.837  0.474 | | 0.105  0.072  -0.09  0.107  0.04  -0.103  0.057 | | 0.03  0.15  0.067  0.029  0.4160.036  0.247 | | 0.101  0.058  -0.1  0.103  0.045  -0.109  0.04 | | 0.050  0.2560.052  0.045  0.3810.034  0.433 | | 0.205  0.14  0.027  0.176  0.191  -0.086  0.093 | | ***  *  0.588**  ***  0.080  0.059 | | 0.201  0.125  0.009  0.159  0.17  -0.081  0.08 | | ***  0.0150.859  *  **  0.113  0.118 |
| Dendritic cell | HLA-DPB1  HLA-DQB1  HLA-DRA  HLA-DPA1  BDCA-1  BDCA-4  CD11c | 0.445  0.295  0.339  0.375  0.429  0.596  0.488 | ***  ***  ***  ***  ***  ***  *** | | 0.293  0.269  0.317  0.354  0.414  0.577  0.472 | | | ***  ***  ***  ***  ***  ***  *** | | 0.087  -0.049  0.021  0.063  0.308  0.568  0.283 | | 0.0770.316  0.6640.198  ***  ***  *** | | 0.089  -0.061  0.023  0.066  0.323  0.567  0.28 | | 0.0840.236  0.6520.199  ***  ***  *** | | 0.368  0.205  0.297  0.312  0.35  0.687  0.477 | | ***  ***  ***  ***  ***  ***  *** | | 0.343  0.178  0.275  0.287  0.334  0.68  0.462 | | ***  **  ***  ***  ***  ***  *** |
| ***Treg*** | FOXP3  CCR8  STAT5B | 0.468  0.474  0.465 | ***  ***  *** | | 0.452  0.464  0.462 | | | ***  ***  *** | | 0.142  0.291  0.465 | | *  ***  *** | | 0.136  0.297  0.458 | | *  ***  *** | | 0.281  0.376  0.531 | | ***  ***  *** | | 0.26  0.369  0.53 | | ***  ***  *** |

*Continued*

| **Description** | **Gene markers** | **STAD-TGFβ1** | | | | | | |  | | **STAD-TGFβ2** | | | | | |  | | **STAD-TGFβ3** | | | | | |
| --- | --- | --- | --- | --- | --- | --- | --- | --- | --- | --- | --- | --- | --- | --- | --- | --- | --- | --- | --- | --- | --- | --- | --- | --- |
|  |  | **None** | |  | | **Purity** | | |  | | **None** | |  | | **Purity** | |  | | **None** | |  | | **Purity** | |
|  |  | **Cor** | ***P*** |  | | | **Cor** | ***P*** |  | | **Cor** | ***P*** |  | | **Cor** | ***P*** |  | | **Cor** | ***P*** |  | | **Cor** | ***P*** |
| Th1 | T-bet  STAT4  STAT1  IFN-γ  TNF-α | 0.447  0.426  0.264  0.161  0.299 | ***  ***  ***  **  *** | | 0.438  0.392  0.262  0.151  0.273 | | | ***  ***  ***  *  *** | | 0.136  0.283  0.119  -0.027  0.135 | | *  ***  0.0150.585  * | | 0.145  0.274  0.109  -0.023  0.137 | | *  ***  0.0340.654  * | | 0.323  0.372  0.155  0.096  0.14 | | ***  ***  *  0.051* | | 0.306  0.349  0.147  0.088  0.1 | | ***  ***  *  0.0870.052 |
| Th2 | GATA3  STAT6  STAT5A  IL13 | 0.551  0.196  0.465  0.177 | ***  ***  ***  ** | | 0.54  0.203  0.467  0.93 | | | ***  ***  ***  ** | | 0.282  0.134  0.305  0.091 | | ***  *  ***  0.064 | | 0.296  0.136  0.31  0.1 | | ***  *  ***  0.053 | | 0.391  0.153  0.459  0.177 | | ***  *  ***  ** | | 0.372  0.16  0.46  0.205 | | ***  *  ***  ** |
| Tfh | BCL6  IL21 | 0.511  0.203 | ***  *** | | 0.491  0.188 | | | ***  ** | | 0.459  0.061 | | ***  0.214 | | 0.433  0.066 | | ***  0.2 | | 0.511  0.145 | | ***  * | | 0.492  0.13 | | ***  0.012 |
| Th17 | STAT3  IL17A | 0.356  –0.11 | ***  0.025 | | 0.349  –0.119 | | | ***  0.021 | | 0.411  -0.069 | | ***  0.163 | | 0.398  -0.071 | | ***  0.165 | | 0.411  -0.261 | | ***  *** | | 0.409  -0.269 | | ***  *** |
| T cell exhaustion | PD-1  CTLA4  LAG3  TIM-3  GZMB | 0.488  0.324  0.37  0.503  0.222 | ***  ***  ***  ***  *** | | 0.493  0.307  0.367  0.496  0.193 | | | ***  ***  ***  ***  ** | | 0.135  0.177  0.073  0.217  -0.03 | | *  **  0.135***  0.541 | | 0.148  0.189  0.07  0.226  -0.04 | | *  **  0.173***  0.433 | | 0.273  0.237  0.226  0.505  0.146 | | ***  ***  ***  ***  * | | 0.26  0.22  0.211  0.502  0.116 | | ***  ***  ***  ***  0.024 |

TAM, tumor-associated macrophage; Th, T helper cell; Tfh, Follicular helper T cell; Treg, regulatory T cell; Cor, R value of Spearman’s correlation; None, correlation without adjustment. Purity, correlation adjusted by purity. *P < 0.01; **P < 0.001; ***P < 0.0001.
